# Functional impact of intramolecular cleavage and dissociation of adhesion G protein–coupled receptor GPR133 (ADGRD1) on canonical signaling

**DOI:** 10.1016/j.jbc.2021.100798

**Published:** 2021-05-20

**Authors:** Joshua D. Frenster, Gabriele Stephan, Niklas Ravn-Boess, Devin Bready, Jordan Wilcox, Bjoern Kieslich, Caroline Wilde, Norbert Sträter, Giselle R. Wiggin, Ines Liebscher, Torsten Schöneberg, Dimitris G. Placantonakis

**Affiliations:** 1 Department of Neurosurgery, NYU Grossman School of Medicine, New York, New York, USA; 2 Kimmel Center for Stem Cell Biology, NYU Grossman School of Medicine, New York, New York, USA; 3 Institute of Bioanalytical Chemistry, Center for Biotechnology and Biomedicine, University of Leipzig, Leipzig, Germany; 4 Medical Faculty, Rudolf Schönheimer Institute of Biochemistry, University of Leipzig, Leipzig, Germany; 5 Heptares Therapeutics Ltd, Cambridge, UK; 6 Laura and Isaac Perlmutter Cancer Center, NYU Grossman School of Medicine, New York, New York, USA; 7 Brain and Spine Tumor Center, NYU Grossman School of Medicine, New York, New York, USA; 8 Neuroscience Institute, NYU Grossman School of Medicine, New York, New York, USA

**Keywords:** adhesion GPCR, intramolecular cleavage, NTF–CTF dissociation, glioblastoma, glycosylation, intracellular trafficking, receptor structure–function, signaling, subcellular fractionation, BFA, brefeldin A, BSA, bovine serum albumin, CTF, C-terminal fragment, DDM, n-dodecyl β-D-maltoside, EndoH, endoglycosidase H, ER, endoplasmic reticulum, GBM, glioblastoma, Golgi, Golgi apparatus, GPCRs, G protein–coupled receptors, GPS, GPCR proteolysis site, hPAR1, human protease-activated receptor 1, HTRF, homogenous time-resolved fluorescence, MWs, molecular weights, NTF, N-terminal fragment, Nuc, nucleus, PAR1, protease-activated receptor 1, PM, plasma membrane

## Abstract

GPR133 (*ADGRD1*), an adhesion G protein–coupled receptor (GPCR) whose canonical signaling activates G_αS_-mediated generation of cytosolic cAMP, has been shown to be necessary for the growth of glioblastoma (GBM), a brain malignancy. The extracellular N terminus of GPR133 is thought to be autoproteolytically cleaved into N-terminal and C- terminal fragments (NTF and CTF, respectively). However, the role of this cleavage in receptor activation remains unclear. Here, we used subcellular fractionation and immunoprecipitation approaches to show that the WT GPR133 receptor is cleaved shortly after protein synthesis and generates significantly more canonical signaling than an uncleavable point mutant GPR133 (H543R) in patient-derived GBM cultures and HEK293T cells. After cleavage, the resulting NTF and CTF remain noncovalently bound to each other until the receptor is trafficked to the plasma membrane, where we demonstrated NTF–CTF dissociation occurs. Using a fusion of the CTF of GPR133 and the N terminus of thrombin-activated human protease-activated receptor 1 as a controllable proxy system to test the effect of intramolecular cleavage and dissociation, we also showed that thrombin-induced cleavage and shedding of the human protease-activated receptor 1 NTF increased intracellular cAMP levels. These results support a model wherein dissociation of the NTF from the CTF at the plasma membrane promotes GPR133 activation and downstream signaling. These findings add depth to our understanding of the molecular life cycle and mechanism of action of GPR133 and provide critical insights that will inform therapeutic targeting of GPR133 in GBM.

The adhesion family of G protein–coupled receptors (GPCRs) has attracted increasing interest in the recent years for essential functions in health and disease (1, 2). The large extracellular N termini of adhesion GPCRs contain a GPCR autoproteolysis-inducing domain, which is thought to catalyze autoproteolytic cleavage at the GPCR proteolysis site (GPS) marked by the tripeptide sequence H-L/I-∗-S/T (∗ denotes the cleavage site) (3, 4). After this intramolecular cleavage, adhesion GPCRs are generally believed to exist as noncovalently bound heterodimers of their extracellular N-terminal fragment (NTF) and transmembrane-spanning C-terminal fragment (CTF) (5, 6). The recent demonstration of a tethered internal agonist, also known as the *Stachel* sequence, immediately C-terminal to the GPS, has given rise to the hypothesis that NTF–CTF dissociation facilitates the conformational changes needed for the *Stachel* sequence to initiate receptor activation (7).

However, the mechanism of receptor activation mediated by autoproteolytic cleavage and NTF–CTF dissociation is not generalizable to all members of the adhesion GPCR family. Indeed, several adhesion GPCRs have not yet been observed to undergo intramolecular cleavage, and cleavage is not necessarily required for their activity (2, 8, 9, 10, 11). In addition, in some adhesion GPCRs, cleavage occurs in selective cellular contexts but not others (4, 12, 13, 14). Finally, cleavage- and *Stachel*-independent signaling have been reported for several adhesion GPCRs (8, 10, 15, 16, 17). These observations emphasize the need to study mechanisms of activation for adhesion GPCRs on an individual basis and in physiologically relevant biological contexts.

We previously described that GPR133 (ADGRD1), a member of the adhesion family of GPCRs, is expressed in, and required for growth of, glioblastoma (GBM), an aggressive primary brain malignancy (18, 19, 20). In heterologous expression systems, N-terminally truncated CTF constructs of GPR133 generate significantly more G protein–mediated signaling than the full-length receptor (7). Nonetheless, there has not been any prior study of the extent of GPR133 cleavage or the NTF–CTF association. Here, we demonstrate that GPR133 is almost entirely cleaved in patient-derived GBM cells and that cleaved GPR133 has a higher basal activity than an uncleavable GPR133 point mutant. While the cleaved CTF and NTF remain noncovalently bound to each other within the secretory pathway, we demonstrate that the NTF dissociates from the CTF once reaching the plasma membrane (PM). Using a fusion protein of the N terminus from human protease-activated receptor 1 (hPAR1) receptor and the CTF of GPR133, we show that acutely induced dissociation of the NTF and thus liberation of the CTF at the PM increases canonical signaling of GPR133. These findings favor an NTF–CTF dissociation model for activation of GPR133 signaling.

## Results

### Uncleavable GPR133 generates less cAMP signaling relative to WT GPR133

**Figure 1:**
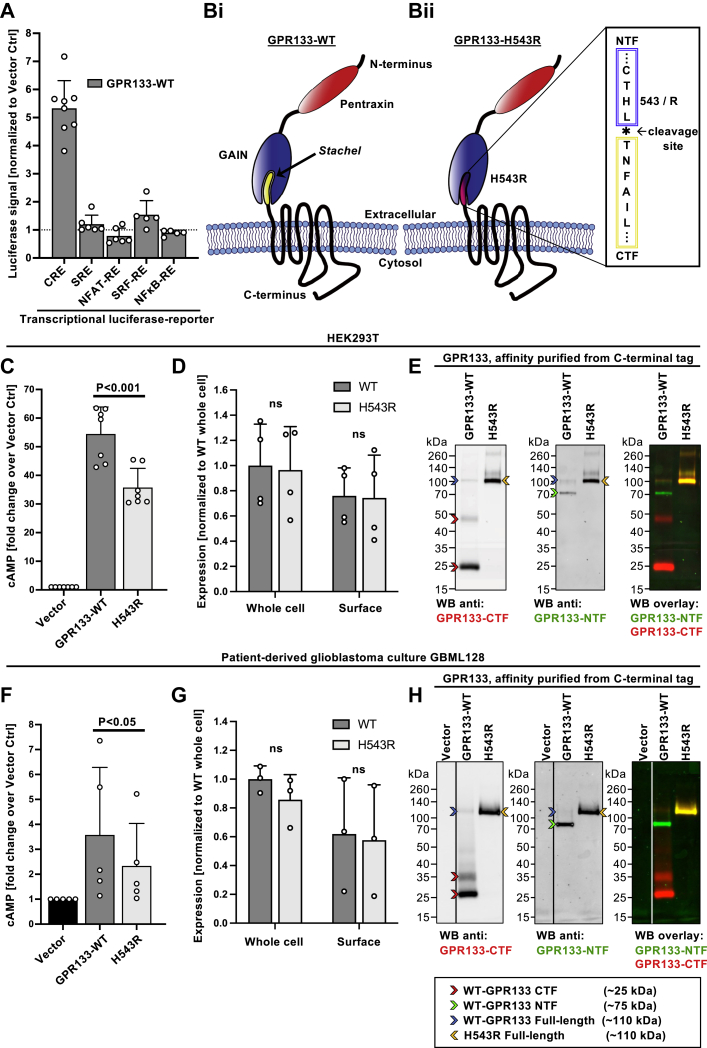
**WT GPR133 is cleaved in patient-derived GBM and HEK293T cells and displays higher cAMP signaling relative to uncleaved GPR133.***A*, HEK293T cells were cotransfected with WT GPR133 and luciferase reporters for various known G protein–mediated signaling pathways. Luciferase signals are expressed as the mean ± SD of normalized fold change over the vector control. GPR133 overexpression in combination with a CRE-Luciferase reporter plasmid confirmed cAMP-mediated signaling as its main canonical signaling pathway (Luciferase fold change over vector: CRE: 5.33 ± 0.99; SRE: 1.21 ± 0.32; NFAT: 0.79 ± 0.28; SRF-RE: 1.54 ± 0.50; NFκB: 0.89 ± 0.10; ANOVA: F_(4, 25)_ = 76.45; *p* < 0.0001; n = 5–8 per reporter). *B*, cartoon schematics of WT (*B*i) and H543R point mutant GPR133 (*B*ii). After cleavage, WT GPR133 is a noncovalently bound heterodimer between the membrane-tethered C-terminal fragment (CTF) and the extracellular N-terminal fragment (NTF). The H543R mutation at the −2 residue of the cleavage site prevents intramolecular cleavage and thereby preserves the full-length receptor structure. *C*, overexpression of WT GPR133 in HEK293T cells results in significantly higher intracellular cAMP levels than the H543R mutant GPR133 as assessed by HTRF assays (cAMP fold change over vector control expressed as the mean ± SD: WT = 54.5 ± 9.4; H543R = 35.8 ± 6.7; ANOVA, F_(2,18)_ = 115.7, *p* < 0.0001; Tukey’s multiple comparisons adjusted *p*-value WT *versus* H543R: *p* < 0.001; n = 7 independent experiments with technical triplicates). *D*, whole-cell and surface ELISA confirms that expression levels of exogenous WT and H543R mutant GPR133 do not significantly differ from each other in HEK293T cells (two-way ANOVA, receptor construct F_(1,12)_ = 2.17, not significant, whole cell *versus* surface F_(1,12)_ = 0.03, not significant, interaction of factors F_(1,8)_ = 0.003, not significant; Tukey’s multiple comparisons, all not significant, n = 4 independent experiments with technical triplicates). *E*, multiplexed fluorescent Western blot analysis of GPR133 cleavage products indicates near-complete cleavage of WT GPR133 in HEK293T cells. Cells were transfected with WT or H543R-mutated GPR133. GPR133 fragments were purified from whole-cell lysates using a C-terminal Twin-Strep affinity tag to reduce nonspecific background staining. Western blot membranes were costained against the GPR133 CTF (*left panel*, and *red* staining in WB overlay) and the GPR133 NTF (*middle panel*, and *green* staining in WB overlay). Cleaved GPR133 CTF bands (25-kDa monomer, 48-kDa presumed multimer), cleaved GPR133 NTF bands (75 kDa), WT GPR133 uncleaved bands (∼110 kDa), and the uncleaved H543R mutant GPR133 band (∼110 kDa) are highlighted with *red*, *green*, *blue*, and *yellow arrows*, respectively. A representative blot is depicted. Corresponding whole-cell lysate input samples are depicted in Fig. S1*C*. *F*, overexpression of WT GPR133 in patient-derived GBM cells results in significantly higher intracellular cAMP levels than the H543R mutant GPR133 as assessed by HTRF assays (cAMP fold change over vector control expressed as the mean ± SD: WT = 3.58 ± 2.71; H543R = 2.33 ± 1.70; two-tailed ratio paired *t* test *p*-value WT *versus* H543R: *p* < 0.05; n = 5 independent experiments with technical triplicates). *G*, whole-cell and surface ELISA confirms that expression levels of exogenous WT and H543R mutant GPR133 do not significantly differ from each other in patient-derived GBM cells (two-way ANOVA, receptor construct F_(1,8)_ = 3.88, not significant, whole-cell *versus* surface F_(1,8)_ = 0.30, not significant, interaction of factors F_(1,8)_ = 0.08, not significant; Tukey’s multiple comparisons, all not significant, n = 3 independent experiments with technical triplicates). *H*, WT GPR133 is almost entirely cleaved in patient-derived GBM cells while H543R mutant GPR133 remains uncleaved. GBM cells were lentivirally transduced with an empty vector control, WT, or H543R-mutated GPR133 and analyzed as described in panel *E*. A representative blot is depicted. Corresponding whole-cell lysate input samples of this and three additional patient-derived GBM cultures are depicted in Fig. S1*D*. GAIN, G protein-coupled receptor autoproteolysis inducing domain; GBM, glioblastoma; HTRF, homogenous time-resolved fluorescence.

Previous reports suggested canonical signaling by GPR133 is mediated *via* coupling to Gα_S_, resulting in an increase of intracellular cAMP (11). We independently confirmed that expression of GPR133 in HEK293T cells is associated with robust increase in cAMP levels as detected by a cAMP response element–Luciferase reporter, but not other known GPCR signaling pathways (Fig. 1*A*). To test whether intramolecular cleavage has implications for canonical signaling, we generated an H543R mutant GPR133 carrying a point mutation at the −2 residue of the GPS cleavage site (11) (Fig. 1*B*i and ii and Fig. S1, *A* and *B*). This mutation is known to abolish GPR133 cleavage but still permits cAMP signaling (7, 11). We used homogenous time-resolved fluorescence (HTRF) assays to measure the cAMP levels produced by the cleaved WT and uncleaved mutant receptor in HEK293T cells. While overexpression of either receptor variant significantly raised cAMP levels above background, indicating a high baseline signaling activity, overexpression of the uncleavable H543R mutant GPR133 increased cAMP levels only to ∼60% of the cAMP levels obtained with WT GPR133 (Fig. 1*C*). This difference in signaling intensity could not be explained by differences in expression levels of the constructs as assessed by ELISA (Fig. 1*D*). Our previous work has demonstrated that GPR133 is expressed in human GBM (19), as well as our own patient-derived GBM cultures, where it is required for tumor growth (18, 20). We thus sought to confirm our findings in the disease-relevant context of patient-derived GBM cultures and obtained similar results (Fig. 1, *F* and *G*). These findings suggest that autoproteolytic cleavage might promote receptor activation.

### WT GPR133 is cleaved in patient-derived GBM and HEK293T cells

To assess the extent of intramolecular cleavage of GPR133, we overexpressed WT GPR133 and the H543R point mutant in HEK293T cells and analyzed whole-cell lysates by Western blot. Multiplexed fluorescent staining with both a commercial antibody (HPA042395, Sigma) against the GPR133 CTF and our own previously described mouse monoclonal antibody against the GPR133 NTF (18, 19) detected separate and distinct bands for the CTF (∼25 kDa) and the NTF (∼75 kDa), respectively (Fig. S1*C*; red arrows mark the CTF, green arrows mark the NTF). Affinity-purifying the receptor fragments to reduce nonspecific background staining confirmed these distinct CTF and NTF bands with increased clarity (Fig. 1*E*; red arrows mark the CTF, and green arrows mark the NTF). For the WT receptor, we also identified a faint band at 110 kDa (blue arrow), which we hypothesized to represent the uncleaved WT GPR133, as well as a faint band around 48 kDa (red arrow), possibly a dimer of the CTF (Fig. 1*E* and Fig. S1*C*). In contrast, the H543R-mutated GPR133 was detected as a single full-length band (∼110 kDa, yellow arrow), consistent with its cleavage deficiency (Fig. 1*E* and Fig. S1*C*; yellow arrows). Because the extent of intramolecular cleavage of adhesion GPCRs has been reported as cell type specific (4, 12, 13, 14), we next interrogated GPR133 cleavage in our patient-derived GBM cultures. Indeed, the same cleavage pattern was confirmed in four separate patient-derived GBM cell cultures (affinity-purified receptor fragments in Fig. 1*H* and whole-cell lysates in Fig. S1*D*). It is important to comment on the discrepancies between expected and observed molecular weights (MWs) of the uncleaved receptor, NTF, and CTF. The expected MW of the uncleaved receptor without the signal peptide is 93 kDa, whereas those of the NTF and CTF are expected to be 57 kDa (without the signal peptide) and 36 kDa, respectively. The shifts in the observed MW of the uncleaved receptor and NTF are due to glycosylation, as demonstrated later in the article. The shift in the MW of the CTF from 36 kDa (expected) to 25 kDa (observed) is likely explained by increased SDS loading on the helical hydrophobic transmembrane segments of the CTF, as previously reported for other transmembrane proteins (21).

Overall, these findings suggest that GPR133 is almost entirely cleaved in human GBM and HEK293T cells.

### Intramolecular cleavage of GPR133 is not required for subcellular trafficking to the PM

**Figure 2:**
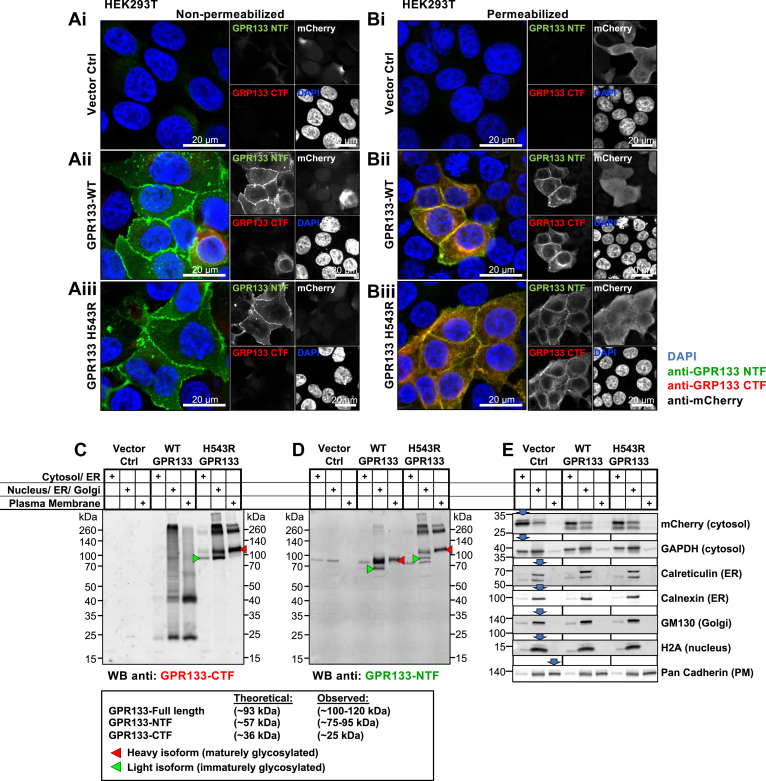
**Intramolecular cleavage of GPR133 is not required for subcellular trafficking to the plasma membrane.***A* and *B*, representative confocal microscopy micrographs of HEK239T cells overexpressing GPR133 show comparable plasma membrane expression patterns for the WT and H543R-mutated GPR133. HEK293T cells were transfected with an empty vector control (*A*i and *B*i), WT GPR133 (*A*ii and *B*ii), or H543R-mutated GPR133 (*A*iii and *B*iii), stained by indirect immunofluorescence under either nonpermeabilizing (*A*) or permeabilizing (*B*) conditions. mCherry is coexpressed on all vectors used in this study and is included in the single-channel panels as transfection control (detected by anti-mCherry antibody staining) but is not included in the merged composite panels. Nuclei were counterstained with DAPI. The scale bars represent 20 μm. *C* and *D*, subcellular fractionation of HEK293T cells expressing an empty vector control (lanes 1–3), WT (lanes 4–6), or H543R-mutated GPR133 (lanes 7–9). A representative Western blot stained against the GPR133 CTF (*C*) and the NTF (*D*) is depicted. Both WT and H543R-mutated GPR133 are detected in the plasma membrane fractions. Lower molecular weight bands of immaturely glycosylated isoforms are highlighted with *green arrowheads* (WT NTF at ∼75 kDa, H543R full-length at ∼100 kDa), higher molecular weight bands of maturely glycosylated isoforms are highlighted with *red arrowheads* (WT NTF at ∼95 kDa, H543R full length at ∼120 kDa). Comparable results were obtained in n = 5 independent experiments. The distribution of the receptor fragments across the subcellular fractions is quantified in Figure 4*C*. *E*, subcellular compartment markers validate the specific enrichment of the subcellular fractions as annotated. Panels *C*–*E* depict the same samples of a representative subcellular fractionation. CTF, C-terminal fragment; DAPI, 4′,6-diamidino-2-phenylindole; NTF, N-terminal fragment.

To understand mechanisms underlying the increased signaling generated by WT GPR133 compared with the uncleavable mutant receptor, we first analyzed their trafficking to the PM through the secretory pathway. Using confocal microscopy and indirect immunofluorescent staining under nonpermeabilizing conditions, we detected both the WT and the H543R-mutated GPR133 at the PM of cells (Fig. 2*A*i–iii and Fig. S2*A*). Similarly, under permeabilizing conditions, the WT and the H543R-mutated GPR133 demonstrated analogous staining patterns in both intracellular organelles of the secretory pathway, as well as at the PM (Fig. 2*B*i–iii and Fig. S2*B*).

To confirm these findings biochemically, we used subcellular fractionation and Western blot analysis to separately interrogate three fractions enriched for (1) cytosol, with some endoplasmic reticulum (ER) contamination, (2) nucleus, ER, and the Golgi apparatus (Nuc/ER/Golgi), and (3) the PM. It is noteworthy that while the first two fractions showed enrichment for distinct subcellular compartments/organelles, the PM fraction was highly specific, as demonstrated by absence of staining for any non-PM compartment markers by Western blot (Fig. 2*E*). Both the cleaved NTF and CTF of the WT receptor, as well as the uncleaved H543R mutant, were prominently detected in the PM fraction (Fig. 2, *C* and *D*, red arrowheads), consistent with our microscopy data. Collectively, these findings suggested that intramolecular cleavage of GPR133 is not required for subcellular trafficking to the PM and that the observed difference in signaling intensities between the cleaved and uncleaved GPR133 variants is not likely to be caused by subcellular trafficking defects.

### Intramolecular cleavage of GPR133 happens early in the secretory pathway and before mature glycosylation

**Figure 3:**
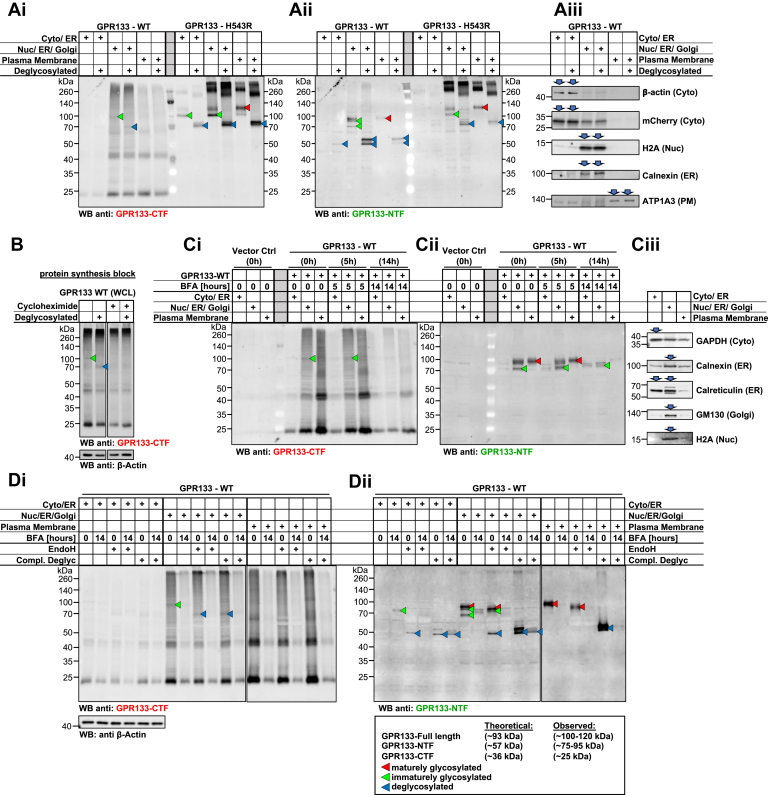
**Intramolecular cleavage of GPR133 happens early in the secretory pathway and before mature glycosylation.** Presumed maturely glycosylated, immaturely glycosylated, and completely deglycosylated forms of GPR133 are marked with *red*, *green*, and *blue arrowheads*, respectively, throughout. *A*, subcellular fractionation of HEK293T cells expressing WT or H543R mutant GPR133 followed by complete enzymatic deglycosylation. GPR133 isoforms were analyzed by Western blot and simultaneously costained with an antibody against the GPR133 CTF (*A*i) and the GPR133 NTF (*A*ii) in separate fluorescent channels. Immaturely and maturely glycosylated isoforms of GPR133 (*green* and *red arrowheads*, respectively) converge at the same molecular weight upon deglycosylation (*blue arrowheads*), as observed in both the full-length GPR133 and the cleaved NTF (*A*i and ii). Note that uncleaved WT GPR133 is detected in the Nuc/ER/Golgi fraction (*A*i, *green* and *blue arrowheads*). Subcellular compartment markers validate enrichment of the respective subcellular fractions (*A*iii). *B*, blocking protein synthesis with cycloheximide abolishes the uncleaved WT GPR133 isoform (*green* and *blue arrowheads* in untreated sample). HEK293T cells overexpressing WT GPR133 were treated with cycloheximide (280 μg/ml) or vehicle control for 8 h. Whole-cell lysates were deglycosylated and analyzed by Western blot using the GPR133 CTF-targeting antibody. β-Actin loading controls in both panels originate from the same membrane and exposure. Representative blots from four independent experiments are depicted. *C*, blocking ER-to-Golgi transport with brefeldin A (BFA) does not result in the accumulation of the uncleaved WT GPR133 isoform (*C*i, *green arrowheads*). GPR133 overexpressing HEK293T cells were treated with 3 μg/ml BFA as annotated, lysed, and subjected to subcellular fractionation. Confocal microscopy micrographs of GPR133 localization after BFA treatment are depicted in Fig. S3*B*. *D*, HEK293T cells overexpressing WT GPR133 were treated with either a dimethyl sulfoxide control or 3 μg/ml brefeldin A (BFA) for 14 h. Whole-cell lysates were subjected to subcellular fractionation followed by treatment with endoglycosidase H (EndoH) or complete enzymatic deglycosylation mix. Note that the uncleaved form of WT GPR133 is sensitive to EndoH treatment (*D*i, *green* and *blue arrowheads*) and that the cleaved NTF exists as both immaturely glycosylated EndoH-sensitive and maturely glycosylated EndoH-insensitive forms (*D*ii). Complete deglycosylation confirms that all observed isoforms of the NTF are the same protein with varying degrees of glycosylation (*blue arrowheads*). Subcellular compartment markers in panel *C*iii validate enrichment of the respective subcellular fractions for samples in panels *C* and *D*. CTF, C-terminal fragment; NTF, N-terminal fragment.

While interrogating the distribution of GPR133 across the subcellular fractions, it became apparent that the cleaved WT GPR133 NTF and the H543R full-length GPR133 undergo an MW shift from lower weight bands in the fractions containing proteins from the early secretory pathway toward higher MW bands in the PM fraction (Fig. 2, *C* and *D*, green and red arrowheads, respectively). Because there are nine N-linked glycosylation sites predicted within the NTF (Fig. S1*A*), we hypothesized that these observed size shifts are due to different extents of glycosylation as the receptor matures through the secretory pathway. To test this hypothesis, we treated the different subcellular fractions with an enzymatic deglycosylation mix (containing PNGase F, O-glycosidase, α2-3,6,8,9 neuraminidase A, β1-4 galactosidase S, and β-N-acetylhexosaminidase_f_). Indeed, upon deglycosylation, the different MW isoforms of cleaved WT NTF and full-length H543R GPR133 shifted to the same predicted MW independent of their subcellular fraction of origin (Fig. 3*A*, green, red, and blue arrowheads demarking immaturely glycosylated, maturely glycosylated, and deglycosylated bands, respectively; deglycosylated whole-cell lysates are shown in Fig. S3*A* for reference), confirming that these bands represent the same protein with different extents of glycosylation.

When staining WT GPR133 using the anti-CTF antibody, we detected a band in the Nuc/ER/Golgi fraction shifting from ∼100 kDa to ∼70 kDa upon deglycosylation (Fig. 3*A*i, green and blue arrowheads, furthest left). This pattern mimics the immaturely glycosylated uncleaved H543R GPR133, leading to our hypothesis that this band is the low-abundance uncleaved form of WT GPR133 we had previously observed in Western blots from whole-cell lysates (Fig. 1, *E* and *H,* and Fig. S1, *C* and *D*). The faster-than-expected mobility of this uncleaved deglycosylated form likely results from increased SDS binding on the helical transmembrane segments of the receptor (21). To test whether this uncleaved and immaturely glycosylated form of WT GPR133 is a stable form of the receptor or a transient state during receptor maturation, we blocked protein synthesis with cycloheximide. Protein synthesis block indeed abolished this uncleaved form of GPR133, supporting the hypothesis that the uncleaved WT GPR133 is a short-lived transition state (Fig. 3*B*, green and blue arrowheads).

To determine whether this short-lived full-length WT GPR133 is cleaved directly after synthesis in the ER, or later in the Golgi, we treated cells with brefeldin A (BFA), which interrupts ER-to-Golgi transport. The effectiveness of BFA to prevent transport along the secretory pathway was confirmed by confocal microscopy (Fig. S3*B*). BFA treatment did not lead to an accumulation of the uncleaved WT GPR133 isoform (Fig. 3*C*i, green arrowheads), suggesting cleavage happens immediately after protein synthesis. However, BFA treatment did result in the elimination of maturely glycosylated NTF in the Nuc/ER/Golgi fraction 14 h after its addition to the medium (Fig. 3*C*ii, red arrowheads).

Finally, we treated the subcellular fractions with endoglycosidase H (EndoH), which removes the immature high mannose glycosylation of proteins within the ER but not mature glycosylation of proteins that have reached the Golgi. Indeed, we observed that the uncleaved WT GPR133 band was sensitive to EndoH-mediated deglycosylation without an additional deglycosylation effect conferred by a complete deglycosylation mix containing PNGase, suggesting that it represents an immaturely glycosylated protein localizing to the ER (Fig. 3*D*).

These data suggest a model in which newly synthesized WT GPR133 carrying immature glycosylation gets intramolecularly cleaved within the ER before trafficking to the Golgi, where it acquires mature glycosylation. WT GPR133 reaches the PM as a fully cleaved and maturely glycosylated protein.

### The GPR133 NTF dissociates from the CTF at the PM

**Figure 4:**
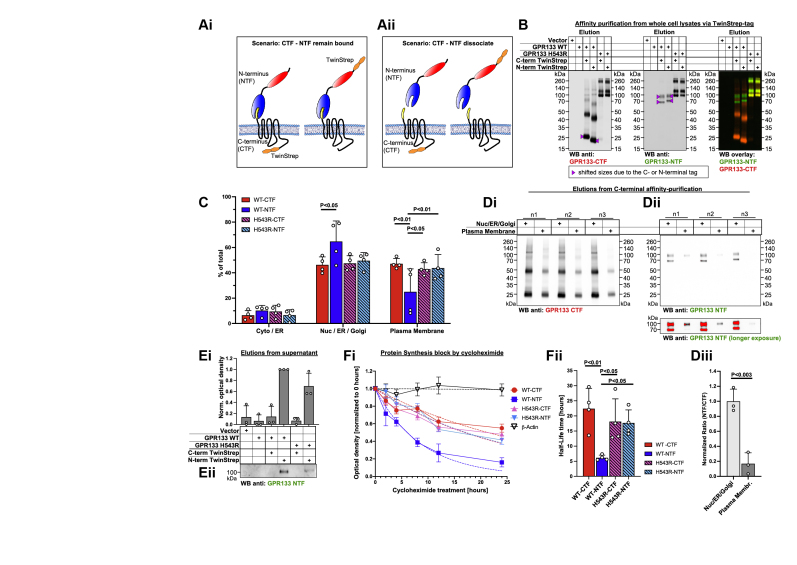
**The GPR133 NTF dissociates from the CTF at the plasma membrane.***A*, schematic of C-terminally and N-terminally tagged GPR133 constructs and experimental rationale. Conceptually, if the CTF and NTF remain bound to each other, receptor constructs with C-terminal or N-terminal affinity tags should purify the CTF and NTF at a one-to-one ratio (*A*i). In the event of dissociation, the location of the affinity tag would bias the ratio of CTF or NTF in the elution (*A*ii). *B*, Western blot analysis of GPR133 constructs affinity-purified from whole-cell lysates. On a whole-cell lysate level, both the C-terminally and N-terminally tagged GPR133 constructs copurify the CTF and NTF at similar stoichiometry, indicating the CTF and NTF to be mostly bound as a heterodimer. *C*, quantified distribution of GPR133 fragments across different subcellular fractions detects relatively less WT GPR133 NTF than CTF at the plasma membrane. HEK293T cells overexpressing either WT or H543R-mutated GPR133 were subjected to subcellular fractionation, followed by Western blotting. The subcellular distribution of each receptor fragment as detected by the NTF- or CTF-targeting antibody was quantified by densitometry. Results are depicted as the mean ± SD (two-way ANOVA, receptor fragment F_(3,48)_ = 3.41 × 10^−8^, *p* > 0.99, subcellular fraction F_(2,48)_ = 153.4, *p* < 0.0001, interaction of factors F_(6,48)_ = 5.02, *p* < 0.001; Tukey’s multiple comparisons Nuc/ER/Golgi WT-CTF *versus* WT-NTF *p* < 0.05; plasma membrane WT CTF *versus* WT NTF *p* < 0.003, WT NTF *versus* H543R CTF *p* < 0.03, WT NTF *versus* H543R NTF *p* < 0.01, n = 5 independent experiments). A representative Western blot membrane of this subcellular distribution is depicted as part of Figure 2, *C* and *D*. *D*, tagged GPR133 CTF copurifies less NTF from the plasma membrane fraction than from the secretory pathway. HEK293T cells overexpressing C-terminally tagged WT GPR133 were subjected to subcellular fractionation, followed by affinity purification of the GPR133 CTF. Elutions from the Nuc/ER/Golgi and the plasma membrane fractions were analyzed by Western blot (*D*i and ii), and intensities were quantified by densitometry. *D*iii, summary of quantified NTF/CTF ratios from *D*i and ii depicted as the mean ± SD. (Nuc/ER/Golgi: 1.00 ± 0.16; plasma membrane: 0.17 ± 0.15; unpaired *t* test *p* < 0.003; n = 3 independent experiments). *Upper* and *lower panels* of *D*ii depict the same membrane at different exposures. *Red overlay* indicates signal saturation. *E*, soluble NTF is detected in cell culture supernatants. Supernatants from HEK293T cells overexpressing different variants of GPR133 were collected and precleared by centrifugation. Tagged receptor fragments were affinity-purified and analyzed by Western blot (*E*ii). The quantified relative densities are depicted as the mean ± SD (*E*i). No staining for the receptor CTF was detected in any condition. Corresponding elutions from whole-cell lysates of the overexpressing cells are depicted in panel *B*. Full-size Western blot membranes for the CTF and NTF staining and deglycosylation of the soluble NTF fragment are depicted in Fig. S4, *A* and *B*. *F*, GPR133 protein half-life and decay curves after protein synthesis block with cycloheximide. HEK293T cells overexpressing either WT or H543R mutated GPR133 were treated with 280 μg/ml cycloheximide and harvested for analysis at different time points. Whole-cell lysates were analyzed by Western blot, and relative abundance of receptor fragments as detected with the CTF- or NTF-targeting antibodies were quantified. *F*i, protein amounts normalized to the beginning of cycloheximide chase are plotted as a function of time. *Dotted lines* are modeled one-phase decay curves used to calculate half-life times. Errors bars denote the SEM. *F*ii, protein half-life times of GPR133 fragments depicted as the mean ± SD. The WT NTF has a significantly shorter half-life in whole-cell lysates than the CTF or the uncleaved receptor (ANOVA F_(3,12)_ = 6.47, *p* < 0.008; Tukey’s multiple comparisons: WT CTF *versus* WT NTF: *p* < 0.006; WT NTF *versus* H543R CTF: *p* < 0.05; WT NTF *versus* H543R NTF: *p* < 0.05; n = 4 independent experiments). Representative Western blots of the cycloheximide time course stained against the CTF and NTF of GPR133 are depicted in Fig. S4*C*i–iv. CTF, C-terminal fragment; NTF, N-terminal fragment.

To investigate whether the cleaved GPR133 CTF and NTF remain noncovalently bound to each other, we created GPR133 constructs carrying either C-terminal or N-terminal Twin-Strep-tags for affinity purification (Fig. 4*A*). When purifying the cleaved receptor from whole-cell lysates, identical stoichiometries of the CTF and NTF were eluted, independent of whether they are purified using the N-terminal or C-terminal affinity tag (Fig. 4*B*).

While this finding suggested that the NTF and CTF remain noncovalently associated at the whole-cell level, we proceeded to dissect whether this association varied depending on the location of GPR133 along the secretory pathway. To this end, we quantified the relative distribution of each receptor fragment (WT CTF, WT NTF, H543R full length as detected with the CTF antibody, H543R full length as detected with the NTF antibody) across the three subcellular fractions mentioned above (Fig. 4*C*). The subcellular distribution did not differ between the cleaved WT CTF and the uncleaved H543R mutant using either the C-terminal or N-terminal antibody, supporting the previous observation that cleavage does not affect the trafficking of the receptor’s transmembrane segment. However, the cleaved WT NTF was significantly underrepresented at the PM when compared with the WT CTF or the H543R uncleaved receptor (Fig. 4*C*). We note that, although the WT NTF appears to be overrepresented in the Nuc/ER/Golgi fraction compared with the WT CTF, this is likely a mathematical artifact arising from reduced representation of the WT NTF in the PM fraction. This finding suggested that either the cleaved NTF traffics within the cells independently of the CTF or, more likely, the NTF dissociates from the CTF at the PM and is thus less abundant in that fraction.

To test these two models, we repeated the purification of the receptor using its C-terminal affinity tag and compared the Nuc/ER/Golgi fraction against the PM fraction as input. We detected a significantly lower NTF-to-CTF ratio at the PM than the Nuc/ER/Golgi fraction (Fig. 4*D*i–iii). Furthermore, we were able to purify and detect the soluble NTF from precleared cell culture supernatants, while not detecting any associated CTFs (Fig. 4*E* and Fig. S4, *A* and *B*). These findings suggested that, while the cleaved NTF and CTF are noncovalently bound in the secretory pathway, they do partially dissociate at the PM.

To gather additional evidence for such NTF–CTF dissociation at the PM, we assayed protein decay of the two fragments after blocking protein synthesis with cycloheximide in HEK293T cells overexpressing either the cleaved WT or uncleaved H543R mutant receptor. Whole-cell lysates of different cycloheximide chase time points were analyzed by Western blot using our CTF- and NTF-targeting antibodies, and intensities were plotted as a function of time (Fig. 4*F*i and ii and Fig. S4*C*). No significant difference was detected between the decay curves and half-life times of uncleaved H543R GPR133 and the WT CTF, indicating that the above described signaling intensity differences were not caused by differences in protein stability. However, the cleaved WT GPR133 NTF decayed at a significantly faster rate than the WT CTF. Although these data could be interpreted as accelerated degradation of the NTF compared with the CTF, they more likely suggest loss of the NTF from whole-cell lysates because of its dissociation and diffusion into the supernatant. This latter interpretation of the soluble NTF is supported by the aforementioned fact that the NTF was detected in precleared cell culture supernatants (Fig. 4*E*) and the fact that we did not detect exogenous NTFs on the surface of cells adjacent to GPR133-overexpressing cells (Fig. S4, *D* and *E*). Therefore, this difference in decay curves offers additional support to the hypothesis that the NTF and CTF time-dependently dissociate at the PM.

### NTF dissociation at the PM increases canonical signaling of a hybrid PAR1–GPR133 receptor

**Figure 5:**
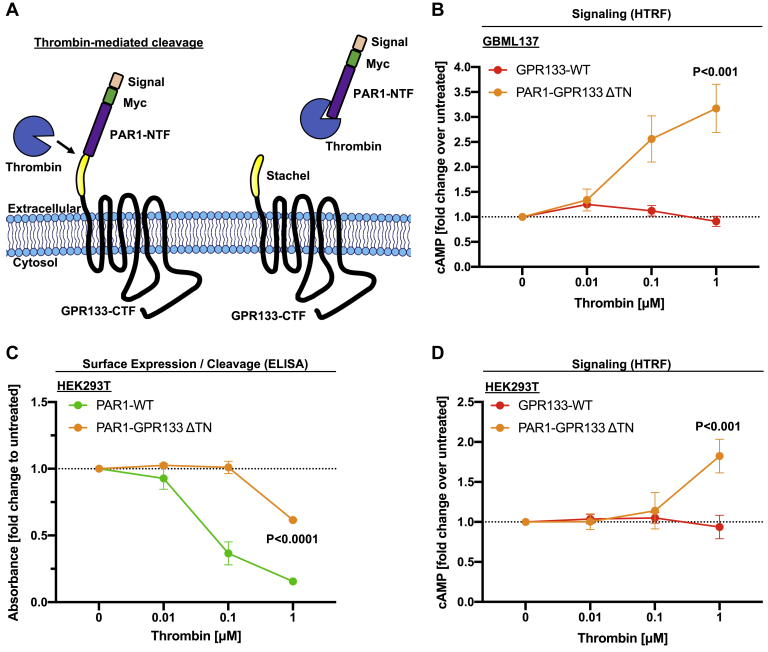
**NTF shedding at the plasma membrane increases canonical signaling of a hybrid PAR1–GPR133 receptor.***A*, cartoon schematic of thrombin-mediated cleavage of the PAR1–GPR133 fusion protein. The Myc-tagged hPAR1 NTF including its thrombin recognition site and subsequent two amino acids (SF) were fused to the GPR133 CTF. The last two amino acids of the PAR1 NTF replaced the first three residues of the GPR133 *Stachel* sequence (TNF→SF, called “ΔTN”). Successful thrombin-mediated cleavage of this construct was assessed by loss of the N-terminal Myc-tag. Detailed information on this and additional fusion constructs is shown in Fig. S5. *B*, patient-derived GBM cultures overexpressing either WT GPR133 or the PAR1–GPR133–ΔTN fusion were exposed to varying concentrations of thrombin, and intracellular cAMP levels were assessed *via* HTRF assays. Data are depicted as the mean ± SEM normalized to the untreated condition. Thrombin-mediated cleavage of the NTF significantly increased canonical GPR133 signaling in the PAR1–GPR133–ΔTN fusion but not WT GPR133 lacking the thrombin recognition site (GBML137: two-way ANOVA, GPR133 constructs F_(1,16)_ = 27.73, *p* < 0.0001, thrombin F_(3,16)_ = 7.17, *p* < 0.003, interaction of factors F_(3,16)_ = 9.27, *p* < 0.001; Tukey’s multiple comparisons, 1 μM thrombin, GPR133 WT *versus* PAR1–GPR133–ΔTNF: *p* < 0.001, n = 3 independent experiments with technical triplicates). *C*, surface ELISA detects the thrombin-mediated cleavage of the Myc-tagged PAR1–NTF in both the full-length PAR1 (positive control) and the PAR1–GPR133–ΔTN fusion protein in a concentration-dependent manner in HEK293T cells (Tukey’s multiple comparisons, PAR1–GPR133–ΔTN fusion 1 μM thrombin *versus* untreated: *p* < 0.0001, n = 3 independent experiments with technical triplicates). *D*, HEK293T cells overexpressing either WT GPR133 or the PAR1–GPR133–ΔTN fusion were exposed to varying concentrations of thrombin, and intracellular cAMP levels were assessed *via* HTRF assays. Data are depicted as the mean ± SEM normalized to the untreated condition. Thrombin-mediated cleavage of the NTF significantly increased canonical GPR133 signaling in the PAR1–GPR133–ΔTN fusion, but not WT GPR133 lacking the thrombin recognition site (two-way ANOVA, GPR133 constructs F_(1,16)_ = 6.61, *p* < 0.03, thrombin F_(3,16)_ = 3.67, *p* < 0.04, interaction of factors F_(3,16)_ = 5.68, *p* < 0.008; Tukey’s multiple comparisons, 1 μM thrombin, GPR133 WT *versus* PAR1–GPR133–ΔTN: *p* < 0.004, n = 3 independent experiments with technical triplicates). Absolute values and additional variations of the PAR1–GPR133 fusion constructs are shown in Fig. S5. CTF, C-terminal fragment; hPAR1, human protease-activated receptor 1; HTRF, homogenous time-resolved fluorescence; NTF, N-terminal fragment.

The data above suggest that the NTF is shed at the PM, which may explain the elevated signaling of cleaved WT GPR133 relative to the uncleavable receptor. To directly test whether NTF dissociation is the mechanism responsible for increased signaling, we constructed a fusion protein between the N terminus of the human protease-activated receptor 1 (PAR1) and the CTF of GPR133, following the design of Mathiasen *et al.* (22). In this fusion protein, the PAR1 NTF serves as a proxy for the NTF of GPR133 by capping the GPR133 CTF during maturation, while also adding a thrombin recognition site for enzymatically inducible cleavage and release of the NTF at the PM (Fig. 5*A* and Fig. S5*A*). At the cleavage side of this fusion protein, the residues “SF” of PAR1 replace the residues “TNF” of GPR133’s *Stachel* region, thus labeled “PAR1–GPR133 ΔTN” (fusion construct and tested alternative designs detailed in Fig. S5). Using patient-derived GBM cells, we indeed observed that thrombin-mediated cleavage of the fusion protein resulted in a significant increase in intracellular cAMP levels in a concentration-dependent manner (Fig. 5*B* and Fig. S5*B*i and ii). WT GPR133 lacking the thrombin recognition site did not respond to thrombin treatment, supporting the specificity of this effect (Fig. 5*B* and Fig. S5*B*i and ii). Similar results were obtained in HEK293T cells (Fig. 5*D* and Fig. S5*D*i and ii). Using cell surface ELISA in HEK293T cells, we demonstrated that this exposure to thrombin indeed led to the dissociation of the PAR1–NTF from the GPR133–CTF, with a concentration dependence that paralleled our signaling data (Fig. 5*C* and Fig. S5*C*i and ii). These findings support the hypothesis that NTF dissociation and thus liberation of the GPR133 CTF promotes full activation of GPR133.

## Discussion

Classically, GPCRs are thought to exist in an equilibrium between an active and inactive state. Upon encountering a stimulus, such as ligand binding, this equilibrium shifts toward the “on” state by stabilizing the receptor in a certain conformation (23). For adhesion GPCRs, such a shift in the equilibrium is hypothesized to be mediated by a tethered internal agonist, the *Stachel* sequence, which resides in the most N-terminal region of the CTF after cleavage (24, 25). There are two possible modes for how the *Stachel* sequence might exert its agonistic effect on adhesion GPCR signaling. Either a conformational change within the extracellular region containing the *Stachel* is sufficient for receptor activation, as would be expected from a classical GPCR, or dissociation of the NTF from the CTF causes unmasking of the *Stachel* sequence, which in turn causes receptor activation. The facts that uncleavable mutant adhesion GPCRs in some cases phenocopy the canonical signaling of their WT counterparts, and that several adhesion GPCRs are not cleaved, argue for the former model (8, 10, 15). However, examples of cleavage-deficient mutants manifesting reduced signaling capacity (26, 27, 28, 29), reports of soluble NTFs of adhesion GPCRs detected *in vitro* and *in vivo* (9, 29, 30, 31), and examples of deletion mutants mimicking CTFs that demonstrate increased signaling relative to their WT counterparts (7, 15, 32) argue in favor of the latter model. In the case of GPR133, where the uncleavable H543R mutant demonstrates 60% of the basal activity of the WT cleaved receptor, it is possible that the *Stachel* sequence is already prebound in the agonist-binding site of the CTF and cleavage allows full isomerization to the active state. Such a model would be consistent with isomerization properties of other GPCRs with tethered agonists (33, 34, 35).

**Figure 6:**
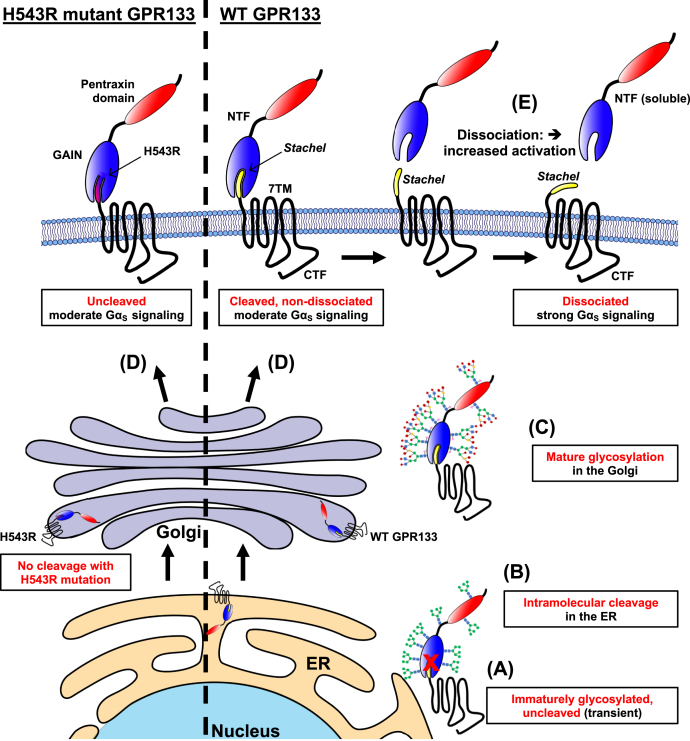
**Proposed model of GPR133’s molecular life cycle.***A*, newly synthesized GPR133 in the endoplasmic reticulum (ER) is immaturely glycosylated and uncleaved. *B*, WT GPR133 is intramolecularly cleaved in the ER before mature glycosylation. *C*, GPR133 in the Golgi apparatus gains mature glycosylation on its NTF resulting in a large MW shift by Western blot analysis (Fig. 3 and Fig. S3). The cleaved CTF and NTF remain noncovalently bound to each other as a heterodimer and copurify at a one-to-one ratio. *D*, fully cleaved WT GPR133 and uncleaved H543R-mutated GPR133 indistinguishably traffic to the plasma membrane in a maturely glycosylated state. *E*, the GPR133 NTF dissociates from the CTF resulting in its relatively lower abundance at the plasma membrane and its consequent detection in the supernatant. Acute liberation of the CTF and exposure of the *Stachel* increases canonical signaling of GPR133. CTF, C-terminal fragment; NTF, N-terminal fragment.

Our study provides evidence that intramolecular cleavage of GPR133 further increases receptor activity *via* dissociation of the cleaved N terminus at the PM (Fig. 6). The survey of both patient-derived GBM cells and HEK293T cells indicated that GPR133 is almost entirely cleaved before the receptor reaches the PM. While Western blot analysis of whole-cell lysates did detect a small amount of uncleaved GPR133, we believe that this form of GPR133 represents a transient state of the newly synthesized receptor in the ER. This view is supported by the observations that the uncleaved receptor (1) did not appear in the subcellular fraction representing the PM (Figs. 2*C* and 3, *A*, *C* and *D*); (2) was sensitive to deglycosylation by EndoH, without additional deglycosylation effect conferred by PNGase (Fig. 3*D*); (3) did not accumulate upon BFA treatment (Fig. 3*C*); and (4) was abolished by blocking protein synthesis with cycloheximide (Fig. 3*B*). Our observations are in agreement with previous reports on the adhesion GPCRs CIRL (ADGRL1) and GPR116 (ADGRF5) (36, 37).

Although the intramolecular cleavage of GPR133 takes place early in the secretory pathway, it is not required for glycosylation of the N terminus and subcellular trafficking of GPR133 to the PM. Previous literature on this subject has remained controversial, as it contains examples of both appropriate and arrested trafficking of cleavage-deficient point mutant adhesion GPCRs (4, 15, 29, 36, 38, 39, 40). This controversy raises the question whether the requirement of cleavage for correct subcellular trafficking is truly as receptor-specific as it appears from the literature, or whether misfolding due to the introduced mutations at the cleavage site is responsible for the lack of PM localization in some of the adhesion GPCRs.

Our biochemical analysis indicated that the cleaved NTF and CTF of GPR133 remain noncovalently bound during receptor trafficking, until the NTF dissociates at the PM. We propose that this dissociation underlies the higher signaling capacity of cleaved GPR133 relative to the uncleavable H543R mutant receptor. The conclusion that the dissociation occurs at the PM is supported by the following observations: (1) while on a whole-cell lysate level the CTF and NTF appeared to copurify at similar stoichiometries, suggesting noncovalent association (Fig. 4*B*), the CTF from the PM fraction was significantly less associated with the NTF when compared with the CTF from the ER fraction (Fig. 4*D*i–iii); (2) the NTF displayed a faster decay curve than the CTF in whole-cell lysates after protein synthesis block, indicating that the CTF and NTF are capable of behaving as distinct entities (Fig. 4*F*i and ii); (3) we detected the NTF in the cell culture supernatant, consistent with NTF dissociation from the CTF at the PM (Fig. 4*E*i and ii). To our knowledge, this is the first experimental evidence of such NTF–CTF dissociation for GPR133.

To demonstrate that cleavage followed by NTF dissociation is not only correlated to increased canonical signaling but that these two aspects are causally related, we used a recently published model for experimentally controlled shedding of the NTF (22). We generated a fusion protein between the human PAR1 N terminus and GPR133’s CTF and administered thrombin to induce the enzymatic cleavage of the PAR1 NTF and acute dissociation from the CTF. We showed that this acute thrombin-induced NTF shedding at the PM increases the intensity of canonical signaling of the fusion protein, while not affecting WT GPR133 lacking the thrombin-recognition site.

Having demonstrated that GPR133 is intramolecularly cleaved and that the dissociation of the NTF promotes receptor activation and canonical signaling, our future efforts will focus on identifying mechanisms underlying NTF–CTF dissociation at the PM. One hypothesized mechanism of dissociation is that ligand binding confers mechanical shear stress onto the NTF, thereby pulling it off the CTF (41, 42, 43). This model assumes that the strength of the NTF–ligand interaction exceeds that of the NTF–CTF association. An alternative mechanism may involve conformational shifts at the GPCR autoproteolysis-inducing domain after ligand–NTF binding, leading to weakened NTF–CTF association and shedding, without the need for strong mechanical forces. More likely, a combination of the two scenarios might take place and thus increase the specificity of an activating signal. Other physicochemical parameters may also regulate this dissociation, including pH, stiffness of the extracellular matrix, or receptor multimerization.

In conclusion, we demonstrate that dissociation of the cleaved NTF at the PM increases GPR133 activity and canonical signaling. The fact that the uncleavable GPR133 point mutant is also capable of signaling, albeit to a lesser extent relative to the WT receptor, suggests that the steady-state equilibrium allows for a moderate amount of baseline signaling independent of additional stimuli, possibly due to the endogenous *Stachel* sequence being internally prebound in a configuration permissive for signaling. Alternatively, partial activation of the uncleaved receptor may occur through a combination of ligands, mechanical forces, or other stimuli that intermittently shift the receptor equilibrium to the “on” state. The possibility of extracellular ligands reversibly activating canonical signaling in the absence of intramolecular cleavage has previously been reported for the adhesion GPCR GPR56 (ADGRG1) (16). However, for the WT GPR133, we postulate that dissociation of the NTF irreversibly activates the CTF to the “on” state until the receptor is internalized, degraded, or desensitized. Our findings have implications for basic adhesion GPCR biology and also help elucidate the function of GPR133 in GBM.

## Experimental procedures

### Cell culture

Patient-derived GBM cultures were established and maintained as we previously described (18, 44, 45). In brief, fresh operative specimens were obtained from patients undergoing surgery for resection of GBM after informed consent (NYU IRB study 12-01130). Specimens were mechanically minced using surgical blades followed by enzymatic dissociation using Accutase (Cat# AT104, Innovative Cell Technologies). Cells were either long-term maintained in spheroid suspension cultures on untreated cell culture dishes or grown as attached cultures on dishes pretreated with poly-L-ornithine (Cat# P4957, Sigma) and laminin (Cat# 23017015, Thermo Fisher). The GBM growth medium consisted of Neurobasal medium (Cat# 21103049, Gibco) supplemented with N2 (Cat# 17-502-049, Gibco), B27 (Cat# 12587010, Gibco), nonessential amino acids (Cat# 11140050, Gibco), and GlutaMax (Cat# 35050061, Gibco) and was additionally supplemented with 20 ng/ml recombinant basic Fibroblast Growth Factor (Cat# 233-FB-01M, R&D) and 20 ng/ml Epidermal Growth Factor (Cat# 236-EG-01M, R&D) every other day. Parental tumors of these patient-derived cultures underwent mutational and copy number variation profiling using a focused next-generation sequencing panel of 50 genes (NYU Oncomine focus assay) (46, 47) on an Ion Torrent S5 instrument. All tumors had a WT isocitrate dehydrogenase (IDH) background.

HEK293T (Cat# 632180, Takara) cells were cultured in Dulbecco's modified Eagle's medium (Cat# 11965-118, Gibco) supplemented with 10% fetal bovine serum (Cat# PS-FB2, Peak Serum) and sodium pyruvate (Cat# 11360070, Gibco).

All cells were cultured in humidified cell culture incubators at 37 °C balanced with 5% CO_2_. Patient-derived GBM cells were cultured at 4% O_2_, while HEK293T cells were cultured at 21% O_2_.

### HTRF signaling assays

HEK293T or patient-derived GBM cells were transfected with overexpression plasmids of interest using Lipofectamine 2000 (Cat# 11668-019, Invitrogen) or Lipofectamine 2000 Stem reagent (Cat# STEM00008, Thermo Fisher), respectively, according to the manufacturer’s protocol. Twenty-four hours after transfection, cells were reseeded onto 96-well plates pretreated with poly-L-ornithine (Cat# P4957, Sigma) and laminin (Cat# 23017015, Thermo Fisher) at a density of 75,000 cells per well. Two days after transfection, the medium was exchanged for 50 μl of fresh medium with 1 mM 3-isobutyl-1-methylxanthine (Cat# I7018-100MG, Sigma-Aldrich), and cells were incubated at 37 °C for an additional 30 min. For the thrombin-mediated cleavage experiments, thrombin (Cat# T9326-150UN, Sigma-Aldrich) was added into the medium mix as part of this 30-min incubation. Cells were lysed, and cAMP levels were measured using the cAMP Gs dynamic kit (Cat# 62AM4PEC, CisBio) on the FlexStation 3 (Molecular Devices) according to the manufacturer’s protocol.

### ELISA

Cells were transfected and reseeded as described for HTRF signaling assays. Forty-eight hours after transfection (and after 30 min of thrombin exposure if applicable), cells were washed with HBSS +Ca^2+^/+Mg^2+^ and fixed with 4% paraformaldehyde (Cat# P6148, Sigma-Aldrich) for 20 min at room temperature (RT). For whole-cell ELISA under permeabilizing conditions, all following steps were conducted in the presence of 0.1% Triton X-100; for surface ELISA under nonpermeabilizing conditions, no detergent was added at any time. Cells were blocked in HEK293T media containing 10% FBS for 1 h at RT. Cells were incubated with primary antibodies diluted in HEK293T medium containing 10% FBS at concentrations indicated for 1 h at RT. After three washing steps with PBS, cells were incubated with horseradish peroxidase–conjugated secondary antibodies diluted 1:1000 in HEK293T medium containing 10% FBS for 1 h at RT. After additional three thorough washes with PBS, cells were overlaid with 3,3′,5,5′- tetramethylbenzidine-stabilized chromogen for 20 min (Cat# SB02, Thermo Fisher) followed by an equal volume of acidic stop solution (Cat# SB04, Thermo Fisher).

### Chemical drug treatments

Cycloheximide (Cat# 239765, Sigma-Aldrich) was administered to the cell culture medium at a final concentration of 280 μg/ml to achieve protein synthesis block. BFA (Cat# 00-4506-51, Invitrogen) was administered to the cell culture medium at a final concentration of 3 μg/ml to block ER-to-Golgi transport. 3-Isobutyl-1-methylxanthine (Cat# I7018, Sigma-Aldrich) was administered to the cell culture medium at a final concentration of 1 mM to block phosphodiesterases during cAMP measurements.

### Luciferase vector signaling assays

HEK293T cells were cotransfected with GPR133 overexpression plasmids and either one of the following luciferase signaling-reporter plasmids: cAMP response element–Luciferase (Cat# E8471, Promega), SRE-Luciferase (Cat# E1340, Promega), SRF-RE-Luciferase (Cat# E1350, Promega), NFAT-RE-Luciferase (Cat# E8481, Promega), and NFκB-RE-Luciferase (Cat# E8491, Promega). Twenty-four hours after transfection, cells were reseeded in black 96-well plates at a density of 75,000 cells per well. Forty-eight hours after transfection, cells were lysed and luciferase activity was detected using the Bright-Glo Luciferase assay system (Cat# E2650, CisBio) and a BioTek Synergy H1 microplate reader according to the manufacturer’s protocol.

### Western blot analysis

Cells were lysed in RIPA buffer (Cat#89900, Thermo) supplemented with Halt protease inhibitor cocktail (Thermo, Cat# 78429) and 1% n-dodecyl β-D-maltoside (DDM) (Cat# BN2005, Thermo) for solubilization of GPR133. After 15 min on ice, lysates were gently sonicated in a water-bath Bioruptor (Cat# UCD-300, Diagenode) at medium power level for eight cycles of 15 s “ON” and 60 s “OFF” at 4 °C to shear the chromatin. Whole-cell lysates were precleared by centrifugation at 15,000*g* for 10 min at 4 °C. Protein concentrations were determined using the DC protein assay kit II (Cat# 5000112, Bio-Rad). Protein lysates were reduced in Laemmli buffer (Cat# 1610747, Bio-Rad) containing β-mercaptoethanol at 37 °C for 30 min but were not boiled to prevent aggregation of the GPR133 transmembrane region. Equal amounts of protein were separated by SDS-PAGE and transferred to 0.2 μm nitrocellulose membranes (Cat# 1620112, Bio-Rad). After blocking the membranes in 2% bovine serum albumin (BSA) in TBS-Tween for 1 h at RT, they were simultaneously incubated with multiple primary antibodies of different species (listed in Table 1) at 4 °C overnight and visualized with up to three simultaneous fluorescent Alexa Fluor Plus–conjugated secondary antibodies. Images were acquired using the iBright FL1000 system (Invitrogen). Densitometric quantification of band intensities was conducted in ImageJ.

**Table 1:** Key reagents

Reagent or resource	Source	Identifier
Antibodies		
Rabbit polyclonal anti-GPR133-CTF	Sigma-Aldrich	Cat# HPA042395
Mouse monoclonal anti-GPR133-NTF	Not commercially available (Bayin *et al.*, 2016) (Frenster *et al.*, 2020)	Clone “8E3E8”
Mouse monoclonal anti-Beta-Actin (AC15)	Thermo Fisher	Cat# AM4302
Goat polyclonal anti-GAPDH	Thermo Fisher	Cat# PA1-9046
Chicken polyclonal anti-mCherry	Abcam	Cat# ab205402
Rabbit polyclonal anti-calnexin	Cell Signaling Technology	Cat# 2433S
Recombinant anti-calreticulin (EPR3924)	Abcam	Cat# ab92516
Recombinant anti-GM130 (EP892Y)	Abcam	Cat# ab52649
Recombinant anti-Pan cadherin (EPR1792Y)	Abcam	Cat# ab51034
Mouse monoclonal anti-histone H2A (L88A6)	Cell Signaling Technology	Cat# 3636S
Mouse monoclonal anti-ATP1A3 (XVIF9-G10)	Thermo Fisher	Cat# MA3-915
Mouse monoclonal anti-Myc-tag (9B11)	Cell Signaling	Cat# 2276S
Rabbit polyclonal anti-FLAG-tag (M2)	Sigma-Aldrich	Cat# F7425
Mouse monoclonal anti-HA-tag	Sigma-Aldrich	Cat# H3663
Secondary donkey anti-mouse Alexa Fluor Plus 488	Thermo Fisher	Cat# A32766
Secondary donkey anti-rabbit Alexa Fluor Plus 555	Thermo Fisher	Cat# A32794
Secondary donkey anti-goat Alexa Fluor 647	Thermo Fisher	Cat# A21447
Secondary donkey anti-rabbit Alexa Fluor 647	Thermo Fisher	Cat# A31573
Secondary goat anti-chicken IgY Alexa Fluor Plus 555	Thermo Fisher	Cat# A32932
Secondary chicken anti-mouse HRP-conjugated	Thermo Fisher	Cat# A15975
Chemicals, peptides, and recombinant proteins		
Recombinant human thrombin	Sigma-Aldrich	T9326-150UN
Protein deglycosylation mix II	NEB	P6044
Endoglycosidase Hf	NEB	P0703
Cycloheximide–CAS 66-81-9	Sigma-Aldrich	239765-1ML
Brefeldin A solution (1000X)	Invitrogen	00-4506-51
3-Isobutyl-1-methylxanthine (IBMX)	Sigma-Aldrich	I7018-100MG
DDM (n-dodecyl β-D-maltoside) (10%)	Thermo Scientific	BN2005
Neurobasal medium	Gibco	21103049
EGF	R&D	236-EG-01M
bFGF	R&D	233-FB-01M
GlutaMAX Supplement	Gibco	35050061
MEM nonessential amino acids	Gibco	11140050
N2 supplement	Gibco	17-502-049
B27 supplement	Gibco	12587010
Poly-L-ornithine solution	Sigma-Aldrich	P4957-50ml
Laminin for cell culture	Fisher	23017015
Lipofectamine 2000	Invitrogen	11668-019
Lipofectamine stem reagent (used for GBM cells)	Thermo Fisher	STEM00008
Accutase	Innovative Cell Technologies	AT104
Strep-Tactin Sepharose resin	IBA	2-1201-002
Gravity flow Strep-Tactin Sepharose column	IBA	2-1202-001
10x Buffer E; Strep-Tactin elution buffer with Desthiobiotin	IBA	2-1000-025
10x Buffer R; Strep-Tactin regeneration buffer with HABA	IBA	2-1002-100
10x Buffer W; Strep-Tactin wash buffer	IBA	2-1003-100
BioLock Biotin blocking solution	IBA	2-0205-250
Desthiobiotin	IBA	2-1000-001
Critical commercial assays		
cAMP Gs dynamic kit (HTRF)	CisBio	62AM4PEC
Plasma membrane extraction kit	Abcam	ab65400
Bright-Glo Luciferase assay system	Promega	E2650
ELISA: 3,3′,5,5′-tetramethylbenzidine-stabilized chromogen	Thermo Fisher	SB02
ELISA: stop solution	Thermo Fisher	SS04
Deposited data		
Experimental models: cell lines		
Lenti-X 293T cell line	Takara	632180
Patient-derived GBM cultures	Derived in our lab, not commercially available	GBML61/91/128/137
Recombinant DNA		
CRE-Luciferase (pGL4.29[luc2P/CRE/Hygro])	Promega	E8471
SRE-Luciferase (pGL4.33[luc2P/SRE/Hygro])	Promega	E1340
NFAT-RE-Luciferase (pGL4.30[luc2P/NFAT-RE/Hygro])	Promega	E8481
SRF-Luciferase (pGL4.34[luc2P/SRF/Hygro])	Promega	E1350
NFkB-RE-Luciferase (pGL4.32[luc2P/NFkB-RE/Hygro])	Promega	E8491
Thrombin receptor cDNA ORF clone, human, N-Myc tag	Sino Biological	HG13535-NM
pLVX-EF1alpha-mCherry-N1 vector	Takara	631986
pLVX_EF1a-mCherry_PGK-GPR133-WT	This article	N/A
pLVX_EF1a-mCherry_PGK-GPR133-H543R	This article	N/A
pLVX_EF1a-mCherry_PGK-GPR133-WT-N-terminal_TwinStrep	This article	N/A
pLVX_EF1a-mCherry_PGK-GPR133-WT-C-terminal_TwinStrep	This article	N/A
pLVX_EF1a-mCherry_PGK-GPR133-H543R-N-terminal_TwinStrep	This article	N/A
pLVX_EF1a-mCherry_PGK-GPR133- H543R-C-terminal_TwinStrep	This article	N/A
pLVX_EF1a-mCherry_PGK-HA-GPR133-FLAG	This article	N/A
pLVX_EF1a-mCherry_PGK-GPR133-NTF-H543R-rIgG-Fc-StrepTagII	This article	N/A
pLVX_EF1a-mCherry_PGK-hPAR1-CTF-GPR133-NTF	This article	N/A
pLVX_EF1a-mCherry_PGK-hPAR1CTF-dTN-GPR133-NTF	This article	N/A
pLVX_EF1a-mCherry_PGK-hPAR1CTF-dSF-GPR133-NTF	This article	N/A
pLVX_EF1a-mCherry_PGK-hPAR1CTF-d5-GPR133-NTF	This article	N/A
Software and algorithms		
ImageJ	Schneider *et al.*, 2012	https://imagej.nih.gov/ij/
Phyre2	Kelley *et al.*, 2015	http://www.sbg.bio.ic.ac.uk/phyre2/html/page.cgi?id=index
PyMOL		https://pymol.org/2/

### Subcellular fractionation

Adherent cells were gently scraped off from culture dishes in ice-cold PBS in the absence of digestion enzymes or detergents. Subcellular fractionation was conducted using the PM Protein Extraction kit (Cat# ab65400, Abcam) according to the manufacturer’s protocol with slight variations. All steps are conducted at 4 °C or on ice. In brief, cells were resuspended in the homogenization buffer (Cat# ab65400, Abcam) containing protease inhibitors and were gently broken up using a dounce homogenizer until >90% of cells were ruptured. Homogenates were centrifuged at 700*g* for 10 min, and the supernatant was transferred to a new tube. The pellet containing nuclei and intracellular organelles of the secretory pathway was kept as the “Nuc/ER/Golgi” fraction for this study. The supernatant was centrifuged at 10,000*g* for 30 min. The resulting supernatant was kept as the “cytosol/ER” fraction. The resulting pellet was further purified using the two-phase separation of the PM Protein Extraction kit (Cat# ab65400, Abcam) without alterations to the protocol, resulting in the highly pure “PM” fraction. All fractions were resuspended in 1% DDM (Cat# BN2005, Thermo) for better solubilization of the GPR133 transmembrane region. The genomic DNA of the Nuc/ER/Golgi fraction was sheared using a Bioruptor water bath sonicator (Cat# UCD-300, Diagenode) at medium power level for eight cycles of 15 s “ON” and 60 s “OFF” at 4 °C, and all fractions were precleared by centrifugation at 15,000*g* for 10 min. The different subcellular fractions were then either analyzed by Western blot, used as input for affinity purification, or treated with deglycosylating enzyme mixes.

To determine the subcellular distribution of GPR133 fragments (CTF, NTF, H543R Full-length), Western blots of subcellular fractions from GPR133-overexpressing cells were stained using the antibodies targeting the GPR133 C terminus or N terminus and analyzed by densitometry in ImageJ. Subcellular distribution was calculated as the protein amount of a fragment in one subcellular fraction, divided by the sum of this fragment’s protein amount across all subcellular fractions (percent of total).

### Deglycosylation

Whole-cell lysates, subcellular fractions, or eluted proteins were treated with endoglycosidase Hf (EndoHf, Cat# P0703, NEB) or a complete Protein Deglycosylation Mix II (Cat# P6044, NEB) under denaturing conditions for 16 h at 37 °C as recommended by the manufacturer’s protocol. Heating steps beyond 37 °C were omitted from the protocol to prevent aggregation of the GPR133 transmembrane region.

### Affinity purification

Twin-Strep-tagged GPR133 protein was affinity-purified from whole-cell lysates, subcellular fractions, or cell culture supernatants using Strep-Tactin beads (Cat# 2-1201-002, IBA) according to the manufacturer’s protocols. All steps were performed on ice or at 4 °C. In brief, input proteins in solution containing 1% DDM (Cat# BN2005, Thermo) and protease inhibitor cocktail (Cat# 78429, Thermo) were pretreated with 1/10 volume of 10X Buffer W (Cat# 2-1003-100, IBA) and BioLock (Cat# 2-0205-250, IBA) for 15 min on ice to block free biotin. Solutions were then precleared again by centrifugation at 15,000*g* for 15 min. Proteins were then incubated with Strep-Tactin Sepharose beads (Cat# 2-1201-002, IBA) rotating at 4 °C overnight. The following day, Strep-Tactin beads were pelleted and washed five times in a large excess of 1X Buffer W containing 0.1% DDM and protease inhibitors rotating at 4 °C for 30 min (*e.g.*, 20-μl beads washed with 1 ml buffer). Proteins were eluted from Strep-Tactin beads in three consecutive elutions with 1X Buffer E (Cat# 2-1000-025, IBA) containing 1% DDM and supplemented with desthiobiotin (Cat# 2-1000-001, IBA) to a total concentration of 10 mM. Elutions were pooled and analyzed by Western blot or subjected to deglycosylation as described above.

### Immunofluorescent staining and microscopy

Cells cultured on slides pretreated with poly-L-ornithine (Cat# P4957, Sigma) and laminin (Cat# 23017015, Thermo Fisher) were washed gently with ice-cold PBS and fixed with 4% paraformaldehyde (Cat# P6148, Sigma-Aldrich) for 30 min at RT. For permeabilizing conditions, all following steps were conducted in the presence of 0.1% Triton X-100; for nonpermeabilizing steps, no detergent was added until the nuclear counter staining with 4′,6-diamidino-2-phenylindole. Cells were blocked with 10% BSA in PBS for 1 h at RT and stained with primary antibody mixes in 1% BSA in PBS at 4 °C overnight. Antibodies used are detailed in Table 1. Primary antibody staining was visualized by staining with Alexa Fluor Plus–conjugated secondary antibodies at a concentration of 1:1000 for 1 h at RT. Nuclei were counterstained with 500 ng/ml 4′,6-diamidino-2-phenylindole for 10 min at RT. Confocal laser scanning microscopy was conducted on a Zeiss LSM700, and images were analyzed and exported from ImageJ software.

### Protein decay and half-life analysis

HEK293T cells were transfected with various GPR133 overexpression constructs using Lipofectamine 2000 (Cat# 11668-019, Invitrogen), and each split into multiple 6-well plates after 24 h. Forty-eight hours after transfection, all wells were treated with 280 μg/ml cycloheximide (Cat# 239765, Sigma-Aldrich). The “0-h” control condition was harvested right away, and other conditions were harvested 2, 4, 8, 12, and 24 h later. Cells were washed once with PBS, and whole-cell lysates were prepared and analyzed by Western blot as described above. For each resulting Western blot lane, the amounts of GPR133 fragments as detected by the antibodies targeting the C terminus or N terminus were analyzed by densitometry on ImageJ. Protein amounts of the GPR133 fragments normalized to their “0-h” control conditions were plotted as a function of time in GraphPad Prism (modeled one-phase decay curves) to determine the protein half-life.

### Overexpression plasmids and molecular cloning

All overexpression plasmids are based on the lentiviral vector pLVX-EF1alpha-mCherry-N1 (Cat# 631986, Takara). Codon-optimized cDNA encoding for WT human GPR133 (ADGRD1) was obtained from Axxam and subcloned into the pLVX vector using Gibson Assembly, replacing the puromycin resistance (PuroR) gene. Human PAR1 receptor cDNA was obtained from Sino Biological (Cat#, HG13535-NM). hPAR1–GPR133 fusion constructs as well as all other modifications to the GPR133 overexpression construct were subcloned by Gibson Assembly.

### Structural prediction

The 3D protein structure of GPR133 was predicted and modeled using the Protein Homology/analogY Recognition Engine V 2.0 (Phyre2) web portal (http://www.sbg.bio.ic.ac.uk/∼phyre2/html/page.cgi?id=index) (48). The analysis was run in the “intensive” mode using the following amino acid sequence as the input: MEKLLRLCCWYSWLLLFYYNFQVRGVYSRSQDHPGFQVLASASHYWPLENVDGIHELQDTTGDIVEGKVNKGIYLKEEKGVTLLYYGRYNSSCISKPEQCGPEGVTFSFFWKTQGEQSRPIPSAYGGQVISNGFKVCSSGGRGSVELYTRDNSMTWEASFSPPGPYWTHVLFTWKSKEGLKVYVNGTLSTSDPSGKVSRDYGESNVNLVIGSEQDQAKCYENGAFDEFIIWERALTPDEIAMYFTAAIGKHALLSSTLPSLFMTSTASPVMPTDAYHPIITNLTEERKTFQSPGVILSYLQNVSLSLPSKSLSEQTALNLTKTFLKAVGEILLLPGWIALSEDSAVVLSLIDTIDTVMGHVSSNLHGSTPQVTVEGSSAMAEFSVAKILPKTVNSSHYRFPAHGQSFIQIPHEAFHRHAWSTVVGLLYHSMHYYLNNIWPAHTKIAEAMHHQDCLLFATSHLISLEVSPPPTLSQNLSGSPLITVHLKHRLTRKQHSEATNSSNRVFVYCAFLDFSSGEGVWSNHGCALTRGNLTYSVCRCTHLTNFAILMQVVPLELARGHQVALSSISYVGCSLSVLCLVATLVTFAVLSSVSTIRNQRYHIHANLSFAVLVAQVLLLISFRLEPGTTPCQVMAVLLHYFFLSAFAWMLVEGLHLYSMVIKVFGSEDSKHRYYYGMGWGFPLLICIISLSFAMDSYGTSNNCWLSLASGAIWAFVAPALFVIVVNIGILIAVTRVISQISADNYKIHGDPSAFKLTAKAVAVLLPILGTSWVFGVLAVNGCAVVFQYMFATLNSLQGLFIFLFHCLLNSEVRAAFKHKTKVWSLTSSSARTSNAKPFHSDLMNGTRPGMASTKLSPWDKSSHSAHRVDLSAV. Phyre2 reports 93% of the residues to be modeled with >90% confidence. The resulting pdb file was visualized in PyMOL in a cartoon ribbon format, domains were pseudocolored, and the *Stachel* region was highlighted as a surface model. This predicted structure has not been experimentally validated and only serves as an approximate visual guide.

### Statistical analysis

All statistical comparisons were conducted in GraphPad Prism (v9). Population statistics were represented as the mean ± SD, unless mentioned otherwise. Statistical significance was calculated using Students *t* test and one-way or two-way ANOVA with *post hoc* Tukey’s multiple comparisons test. The threshold of significance was set at *p* < 0.05.

## Data availability

All relevant data are contained within the article.

## Supporting information

This article contains supporting information (22, 48).

## Conflict of interest

D. G. P. and NYU Grossman School of Medicine own a patent in the European Union titled “Method for treating high grade glioma” on the use of GPR133 as a treatment target in glioma. D. G. P. has received consultant fees from Tocagen, Synaptive Medical, Monteris, and Robeaute. G. R. W. is an employee and shareholder of Sosei Heptares.
