# Supplementary material for: Functional impact of intramolecular cleavage and dissociation of adhesion G protein–coupled receptor GPR133 (ADGRD1) on canonical signaling
Source: J Biol Chem. 2021 May 20;296:100798. doi: 10.1016/j.jbc.2021.100798 (PMC8215292; doi:10.1016/j.jbc.2021.100798)

SUPPLEMENTAL FIGURE LEGENDS

Figure S1. Wild-type GPR133 is cleaved in patient-derived GBM and HEK293T cells.

A) 2D domain architecture of GPR133 drawn to scale. GAIN, G protein-coupled receptor (GPCR) autoproteolysis inducing domain; GPS, GPCR proteolysis site; CTF, C-terminal fragment; NTF, N-terminal fragment**.**

B) 3D protein structure prediction for GPR133 modeled by homology using the Phyre2 web portal (http://www.sbg.bio.ic.ac.uk/~phyre2/html/page.cgi?id=index) (48). The *Stachel* region (yellow surface model) is part of the CTF after cleavage but remains enveloped by the GAIN domain of the NTF (blue ribbon model) in the full-length model. This predicted structure has not been experimentally validated. The location of the H543R mutation at the -2 residue of the cleavage site is annotated in this WT structural prediction.

C) Western blot analysis of whole cell lysates from HEK239T cells overexpressing WT or H543R mutant GPR133 (used as inputs to generate **Figure 1E)**. HEK293T cells were transfected with an empty vector control, WT, or H543R mutant GPR133, lysed, and analyzed by Western blot. Membranes were co-stained with an antibody detecting the GPR133 CTF (left panel, red staining in WB overlay) and an antibody against the GPR133 NTF (middle panel, and green staining in WB overlay). The C-terminal antibody detects the cleaved WT GPR133 CTF monomer at ~25 kDa and putative dimer at ~48 kDa (red arrows), as well as an uncleaved WT GPR133 at ~110 kDa (blue arrows). The N-terminal antibody detects the cleaved WT NTF at ~75 kDa (green arrow) as well as the uncleaved WT GPR133 at ~110 kDa (blue arrows). Both the CTF- and NTF-targeting antibodies detect the uncleaved H543R mutant full-length receptor at ~110 kDa (yellow arrows).

D) Four separate patient-derived GBM cultures were transduced via lentivirus (GBML61, 91, 128) or by transfection (GBML137) with either an empty vector control, WT, or H543R mutant GPR133.

Whole cell lysates were analyzed by Western blot using an antibody targeting the GPR133 CTF. In all four cultures, WT GPR133 is detected almost entirely cleaved as CTF band at ~25 kDa (red arrows), while H543R mutant GPR133 is detected as uncleaved band at ~110 kDa (yellow arrows). GBML128 is the corresponding input sample used to generate **Figure 1H**. Membranes were counterstained against β-actin or GAPDH as loading control (lower panels).

Figure S2. WT and H543R mutant GPR133 localize to the plasma membrane.

A, B) Representative confocal microscopy micrographs of HEK293T cells overexpressing GPR133 demonstrate that intramolecular cleavage is not required for plasma membrane localization. Cells were transfected with either a vector control, WT, or H543R mutated GPR133, fixed and fluorescently stained under non-permeabilizing (**A**) or permeabilizing (**B**) conditions against the GPR133 CTF, GPR133 NTF, and transfection marker mCherry (not included in the composite image), as described for **Figure 2**. The fluorescence intensity across all channels was measured along a virtual 2D cross section through the cells (shown by the white dotted line) and plotted as a function of distance in micrometers. Transfected cells demonstrate distinct intensity peaks for GPR133 staining at the plasma membrane for both the WT and the uncleaved H543R mutant GPR133. Nuclei were counterstained with DAPI. Scale bars, 20 μm.

Figure S3. Intramolecular cleavage of GPR133 occurs in the ER and prior to mature glycosylation.

Presumed maturely glycosylated, immaturely glycosylated, and completely deglycosylated forms of GPR133 are marked with red, green, and blue arrowheads respectively, throughout.

A) Whole cell lysates of HEK293T cells overexpressing a vector control, WT, or H543R mutated GPR133 were subjected to complete deglycosylation or control treatment and analyzed by

Western blot. Membrane was counterstained against β-Actin and mCherry as loading controls (lower panels).

B) Representative micrographs of HEK293T cells overexpressing WT GPR133 and treated with DMSO control (**Bi**) or Brefeldin A (BFA) for 14 hours (**Bii**). Cells were fixed, permeabilized, fluorescently stained against the GPR133 NTF, and analyzed by confocal laser scanning microscopy. Untreated control cells demonstrate plasma membrane localization of GPR133, while BFA treated cells demonstrate the brightest GPR133 staining in the perinuclear region. mCherry is co-expressed on all vectors used in this study and is included in the single-channel panels as transfection control but is not included in the composite panels. Nuclei were counterstained with DAPI. Scale bars denote 20 μm.

Figure S4. The GPR133 NTF dissociates from the CTF at the plasma membrane.

A) Soluble GPR133 NTF is detected in precleared cell culture supernatants. Supernatants from HEK293T cells overexpressing various tagged GPR133 constructs were harvested, precleared, and used as inputs for affinity purification. Western blot membranes depict the resulting elutions stained against the GPR133 CTF (**Ai**) and NTF (**Aii**) respectively. Multiple independent repeats are depicted and quantified in main **Figure 4E**.

B) Deglycosylation of the supernatant elutions depicted in **Figure 4Eii** confirms the detected band to be the cleaved GPR133 NTF by molecular weight (~57 kDa after complete deglycosylation).

C) Representative Western blot membranes showing the time-course of GPR133 fragments after blocking protein synthesis with cycloheximide. HEK293T cells overexpressing WT or H543R mutant GPR133 were treated with 280 μg/mL cycloheximide and lysed after varying amounts of time. Resulting whole cell lysates were analyzed by Western blot and GPR133 fragments were quantified by densitometry. The highly stable proteins β-Actin and mCherry were used as loading control (**Civ**).

D) GPR133 is not detected on GBM cells adjacent to ectopic GPR133 overexpressing cells. Patient-derived GBM cultures (GBML91) were sparsely transfected with an ectopic overexpression construct of C- and N-terminally tagged GPR133 (schematic **Di**). Cells were fixed, permeabilized, and stained against the C-terminal FLAG-tag and N-terminal HA-tag of the ectopic GPR133 and analyzed by confocal microscopy. A representative micrograph is depicted in **Dii**. While the tagged ectopic GPR133 NTF was detected on the infected GBM cells (marked by staining for the GPR133-CTF), no additional staining was observed on adjacent cells. Nuclei were counterstained with DAPI. Scale bars, 20 µm.

E) A tagged, secreted form of the GPR133 NTF containing a H543R mutation and the *Stachel* region for structural integrity was overexpressed and analyzed by confocal microscopy as detailed for panel **D**. No ectopic secreted GPR133-NTF is detected on adjacent cells. Heatmap depiction of single channel staining of the tagged NTF is included for higher visual sensitivity. Nuclei were counterstained with DAPI. Scale bars, 20 µm.

Figure S5. NTF shedding at the plasma membrane increases canonical signaling of a hybrid hPAR1-GPR133 receptor.

A) Schematic of the hPAR1-GPR133 fusion construct design. The first two amino acids subsequent to the thrombin recognition site (SF) were reported to be critical for thrombin-mediated cleavage of the human PAR1-NTF. Mathiasen and colleagues proposed to replace the first three amino acids of the *Stachel* sequence (TNF) with these residues in their study on ADGRL1 to create a fusion construct both capable of cleavage and canonical receptor signaling ("∆TN") (Mathiasen et al., 2020). Multiple variations of the fusion between hPAR1-NTF and GPR133-CTF were included in our study. The "∆6" construct is lacking the first 6 residues of the *Stachel* sequence and functions as a negative control. "/" and "*" mark the site of thrombin-mediated cleavage and the endogenous GPR133 cleavage site respectively.

Bi) Patient-derived GBM cells (GBML137) overexpressing either WT GPR133 or the various PAR1-GPR133 fusion were exposed to varying concentrations of thrombin for 30 minutes in the presence of 1 mM IBMX, and intracellular cAMP levels were assessed by HTRF assays. Data is depicted as mean ± SEM of absolute cAMP levels. Thrombin-mediated dissociation of the NTF significantly increased canonical GPR133 signaling in the hPAR1-GPR133-ΔTN fusion construct, but none of the other constructs (Two-way ANOVA: construct $F_{(6,56)}$=7.83, P<0.0001, thrombin $F_{(3,56)}$=0.98, P=0.41, interaction of factors $F_{(18,56)}$=0.79, P=0.69; Tukey's multiple comparisons hPAR1-GPR133-ΔTN 0 µM vs 1 µM thrombin P<0.01; 0.01 µM vs 1 µM thrombin P<0.05; all other comparisons within each construct are not significant; n=3 independent experiments with technical triplicates).

Bii) Data from panel **Bi** normalized to untreated condition expressed as mean ± SEM (Two-way ANOVA: construct $F_{(3,32)}$=27.18, P<0.0001, thrombin $F_{(3,32)}$=7.61, P<0.001, interaction of factors $F_{(9,32)}$=7.32, P<0.0001; Tukey's multiple comparisons hPAR1-GPR133-ΔTN 0 µM vs 0.1 µM thrombin P<0.0001; 0 µM vs 1 µM thrombin P<0.0001; 0.01 µM vs 0.1 µM thrombin P<0.001; 0.01 µM vs 1 µM thrombin P<0.0001; all other comparisons within each construct are not significant; n=3 independent experiments with technical triplicates).

Ci) Cell surface ELISA detects the thrombin-mediated dissociation of the Myc-tagged PAR1-NTF in the full-length PAR1 (positive control) and all PAR1-GPR133 fusion protein variants (Two-way ANOVA: construct $F_{(6,56)}$=117.8, P<0.0001, thrombin $F_{(3,56)}$=27.6, P<0.0001, interaction of factors $F_{(18,56)}$=15,7, P<0.0001; n=3 independent experiments with technical triplicates). Cells were exposed to thrombin for 30 minutes, fixed, and stained against the N-terminal Myc-tag under non-permeabilizing conditions. Of note, the WT GPR133 and the vector control do not contain a Myc-tag and are therefore not detected. ELISA absorbance is displayed in non-normalized arbitrary units as mean ± SEM. **Cii**) Data from panel **Ci** normalized to untreated condition expressed as mean ± SEM (Two-way ANOVA: construct $F_{(4,40)}$=38.7, P<0.0001, thrombin $F_{(3,40)}$=206.0,

P<0.0001, interaction of factors $F_{(12,40)}$=13.8, P<0.0001; n=3 independent experiments with technical triplicates).

Di) HEK293T cells overexpressing either WT GPR133 or the various PAR1-GPR133 fusion constructs were exposed to varying concentrations of thrombin for 30 minutes in the presence of 1 mM IBMX, and intracellular cAMP levels were assessed by HTRF assays. Data is depicted as mean ± SEM of absolute cAMP levels. Thrombin-mediated dissociation of the NTF significantly increased canonical GPR133 signaling in the hPAR1-GPR133-ΔTN fusion construct, but none of the other constructs (Two-way ANOVA: construct $F_{(6,52)}$=30.9, P<0.0001, thrombin $F_{(3,52)}$=0.86, P=0.47, interaction of factors $F_{(18,52)}$=0.76, P=0.78; Tukey's multiple comparisons hPAR1-GPR133-ΔTN 0 μM vs 1 μM thrombin P<0.05; 0.01 μM vs 1 μM thrombin P<0.05; 0.1 μM vs 1 μM thrombin P<0.05; all other comparisons not significant; n=3 independent experiments with technical triplicates).

Dii) Data from panel **Di** normalized to untreated condition expressed as mean ± SEM (Two-way ANOVA: construct $F_{(3,32)}$=5.87, P<0.003, thrombin $F_{(3,32)}$=2.72, P=0.06, interaction of factors $F_{(9,32)}$=3.75, P<0.003; Tukey's multiple comparisons hPAR1-GPR133-ΔTN 0 μM vs 1 μM thrombin P<0.0001; 0.01 μM vs 1 μM thrombin P<0.0001; 0.1 μM vs 1 μM thrombin P<0.0006; all other comparisons within each construct are not significant; n=3 independent experiments with technical triplicates).

Supplementary Figure S1

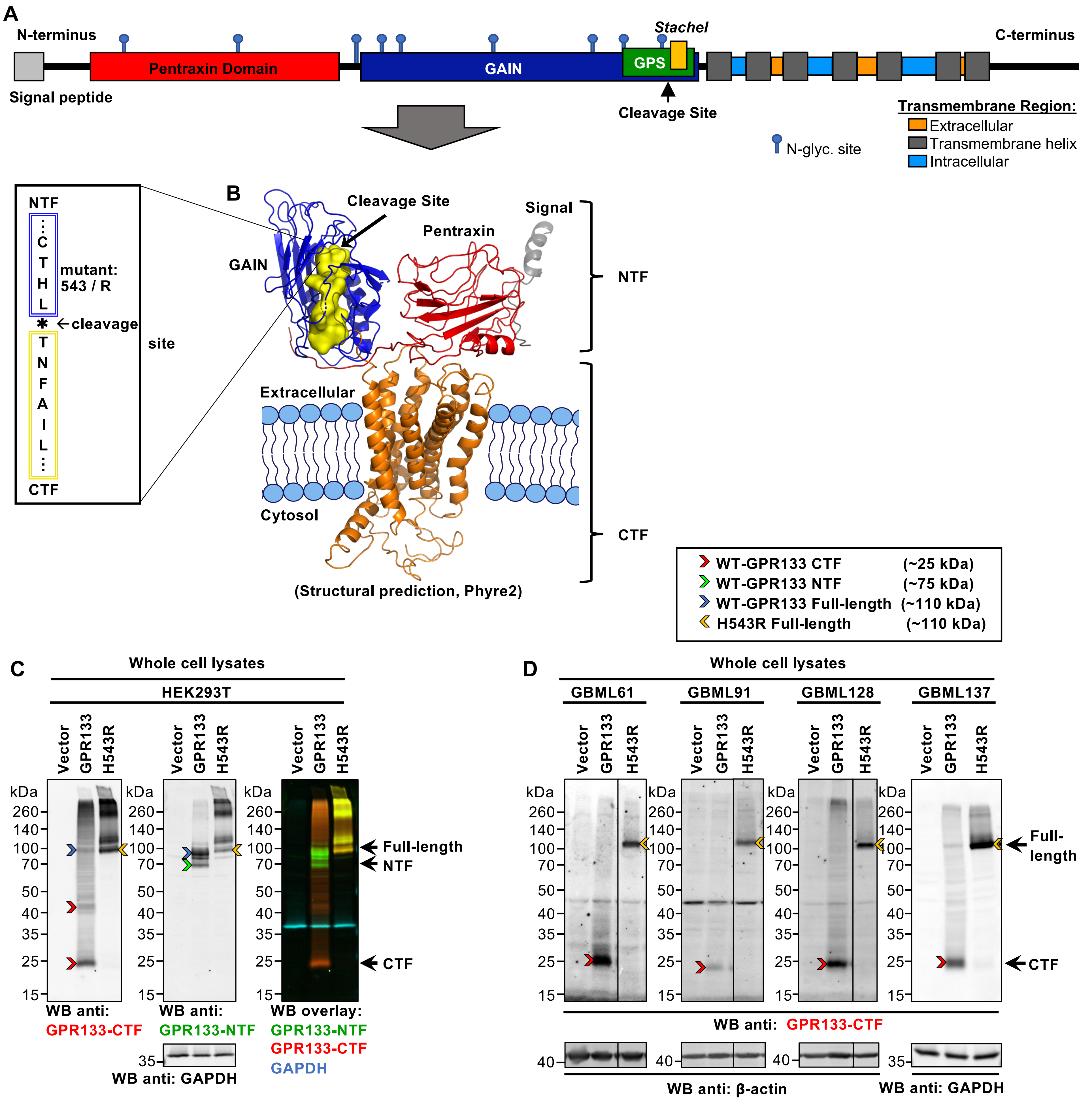

Supplementary Figure S2

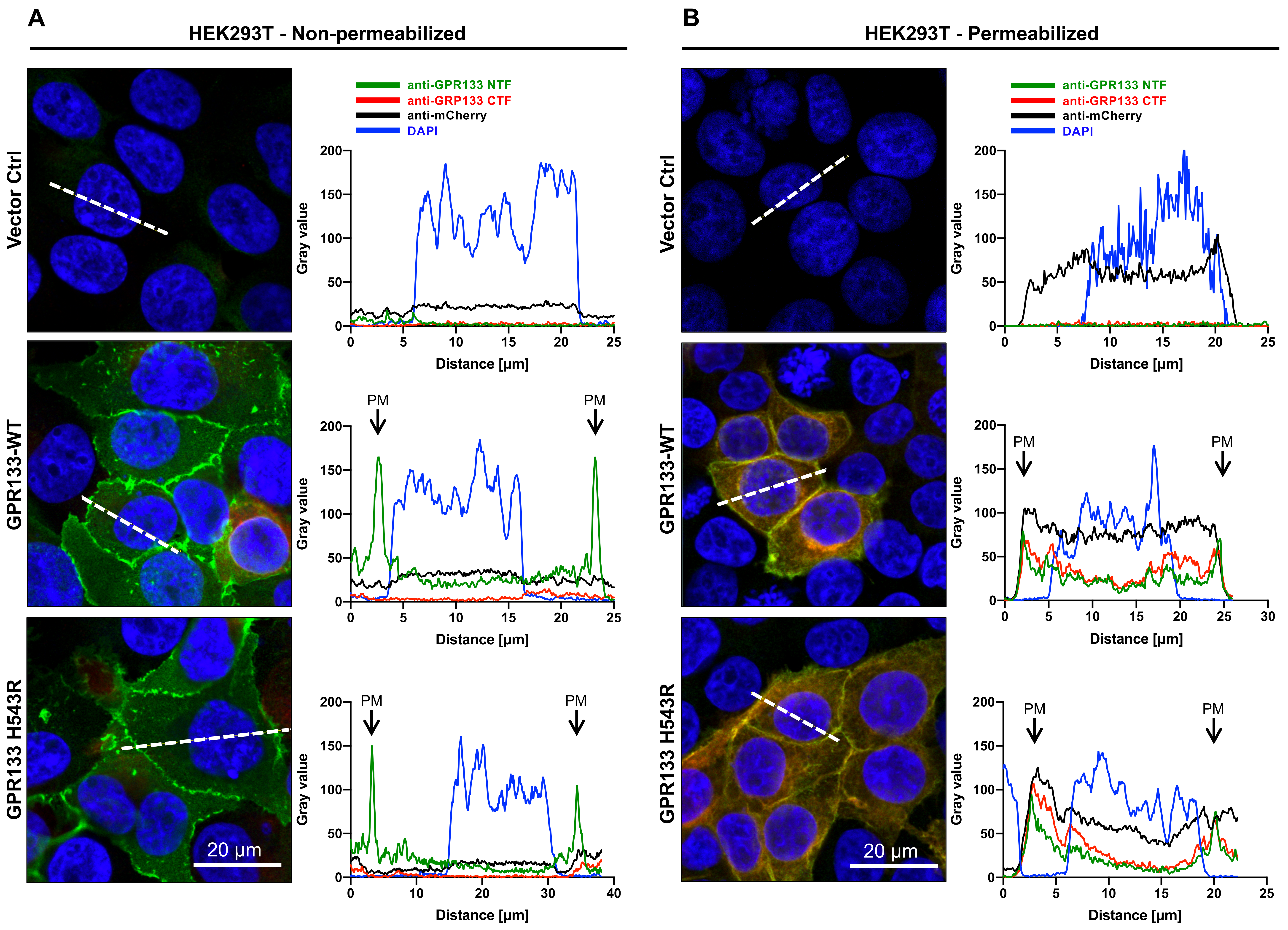

Supplementary Figure S3

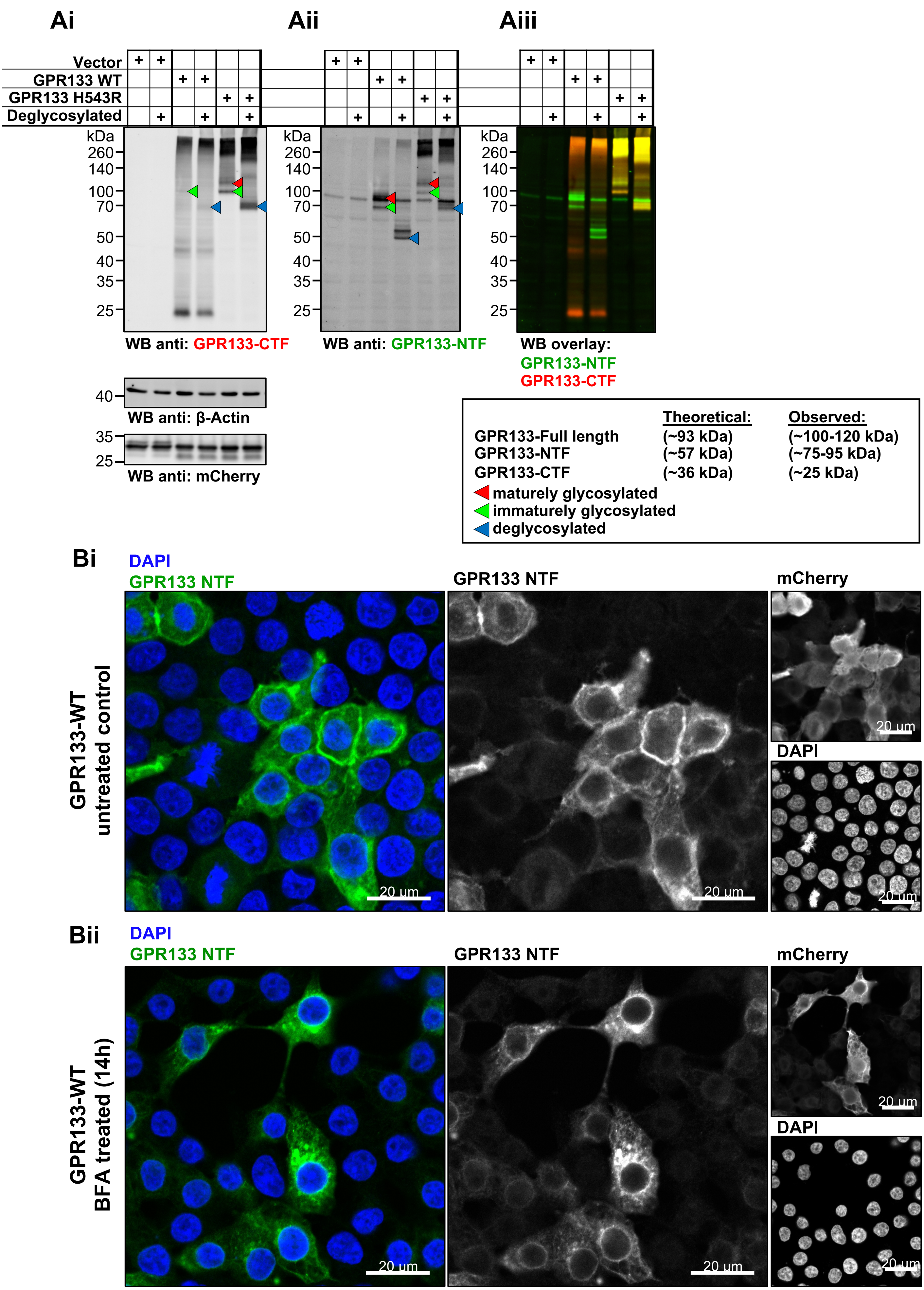

Supplementary Figure S4

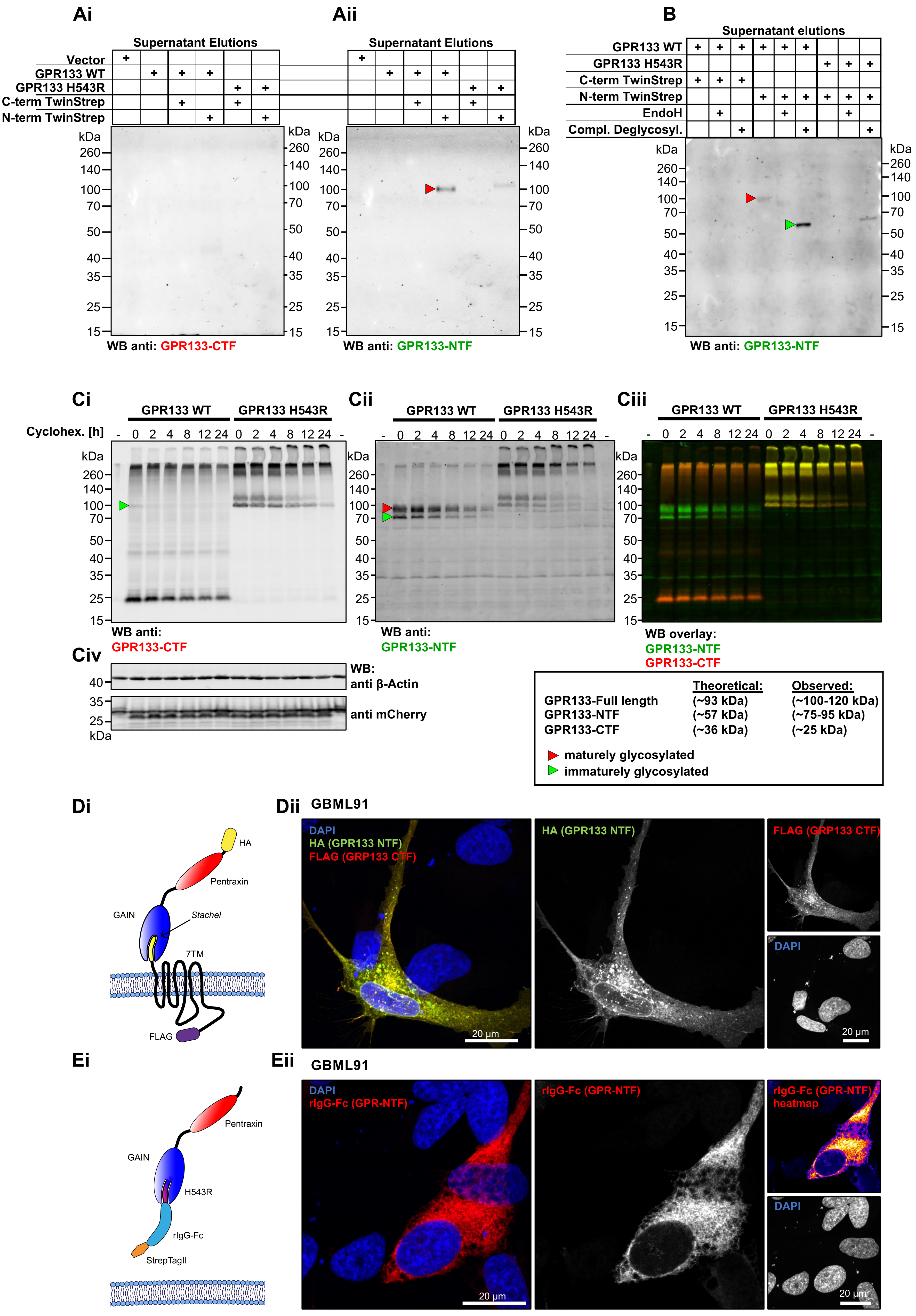

Supplementary Figure S5

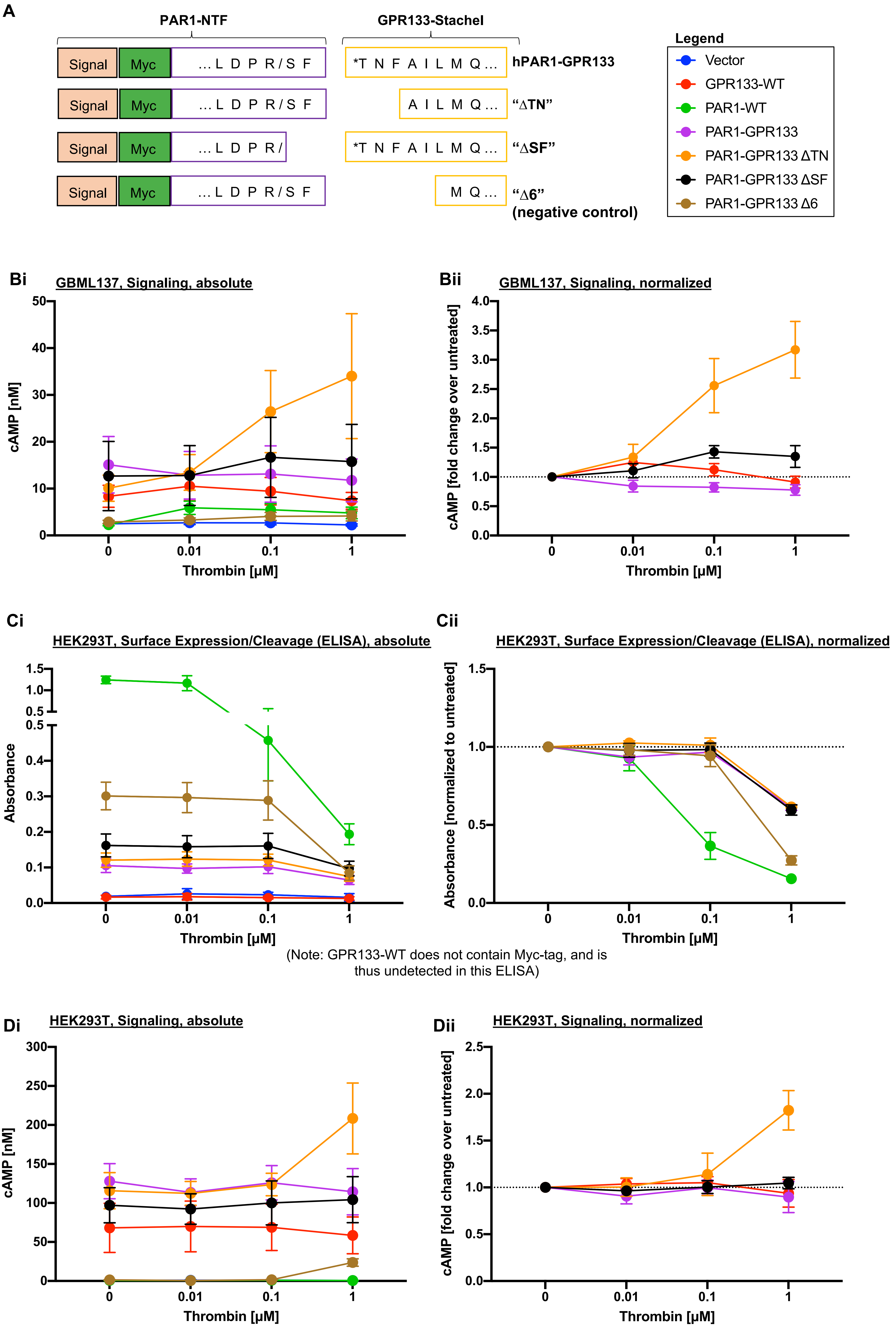

Supplement: Supplemental Figures S1–S5 [file mmc1.pdf]
